# Multi-Scale Landslide Displacement Prediction and Multi-Level Early Warning for the Three Gorges Reservoir Area Using Multi-Source Sensing and Gated-Attention Multimodal Fusion

**DOI:** 10.3390/s26196207

**Published:** 2026-09-30

**Authors:** Liangwu Xu, Xiangjin Ran, Lili Yao, Zhaoji Lin

**Affiliations:** 1 School of Computer Science and Engineering, Sanjiang University, Nanjing 210012, China; yao_lili@sju.edu.cn (L.Y.); lin_zhaoji@sju.edu.cn (Z.L.); 2 College of Earth Sciences, Jilin University, Changchun 130061, China; ranxiangjin@jlu.edu.cn

**Keywords:** multi-source sensing monitoring, Three Gorges Reservoir Area, landslide displacement prediction, multimodal fusion, BiLSTM–Attention, gated-attention mechanism, PSO–Kriging algorithm

## Abstract

Landslides are severe global geological hazards, and landslide displacement prediction is essential for early warning. Focusing on landslides induced by the coupling of periodic reservoir water level fluctuations and local short-term rainstorms in the Three Gorges Reservoir Area (TGRA), existing early-warning methods suffer from coarse rainfall spatial representation, static multimodal-feature fusion, and insufficient deformation-stage-based hierarchical warning mechanisms. Based on multi-source sensing data including Global Positioning System (GPS) displacement monitoring, reservoir water level sensors, ground rain gauges, meteorological radar, and multi-timescale rainfall forecast maps, this study integrates multimodal time-series records of multiple landslide sites from 2007 to 2024. Particle Swarm Optimization–Kriging (PSO–Kriging) interpolation and rainfall-map gridding were employed to construct a dual-source fine-grained rainfall reconstruction scheme. The Bidirectional Long Short-Term Memory (BiLSTM)–Attention network extracted deep temporal features, and a gated-attention fusion mechanism realized 1 d–15 d multi-scale landslide displacement prediction. Combined with the five-stage landslide creep theory, a four-level early-warning system was established. Experimental results show that the proposed multimodal-fusion model achieves a Root Mean Square Error (RMSE) of 3.42 ± 1.27 mm for the 1 d prediction horizon. The model F1-score reaches 89.3 ± 1.9% for short-term warning and 81.7 ± 1.2% for medium-and-long-term warning, which fits well with long-term reservoir-monitoring scenarios. This framework provides feasible technical support for reservoir landslide hazard prevention using multi-source sensing.

## 1. Introduction

Landslides are among the most widespread and destructive geohazards worldwide. They cause tens of thousands of casualties and property losses amounting to billions of US dollars every year [1,2]. The Three Gorges Reservoir Area represents one of the largest reservoir-engineering regions in China and across the globe. Reservoir water levels fluctuate periodically between 145 m and 175 m under reservoir regulation and flood-season rainfall, triggering frequent landslide hazards. Bank-slope stability is critical to the safety of tens-of-millions of local residents [3,4].

Landslide displacement evolution follows classic creep laws, and displacement-time (*S* − *t*) curves serve as an essential basis for multi-level early-warning implementation [5,6,7]. Yin et al. (2010) constructed a landslide real-time monitoring-warning system in the Three Gorges Reservoir and proposed four-level early-warning criteria based on multi-source monitoring data [8]. The wide deployment of monitoring techniques including GNSS, InSAR, and deep-borehole displacement meters has generated abundant fine-grained landslide displacement time-series datasets. These datasets support the development of data-driven landslide displacement prediction models [9]. Landslides within the Three Gorges Reservoir Area show highly complex deformation mechanisms. Their displacement evolution is controlled by multiple interacting factors. These include the intrinsic mechanical properties of rock–soil masses, rainfall infiltration, reservoir water level fluctuation, and groundwater movement [10]. Rainfall acts as the most direct external trigger for reservoir-zone landslides. Periodic reservoir water level variation indirectly modifies slope seepage and stress fields and further influences slope stability [11]. Therefore, it is of great academic significance and practical value to build displacement prediction models with multimodal monitoring-data fusion. Such models should adaptively capture multi-factor synergistic effects for reservoir landslide hazard mitigation and early-warning.

Landslide displacement prediction methodologies have evolved from traditional statistics and machine learning approaches toward deep-learning techniques [12]. Statistical models such as regression analysis and Markov chains are easy to implement, yet they fail to characterize the strong nonlinearity of landslide deformation [13,14]. Machine learning algorithms including random forest have been widely adopted for landslide susceptibility assessment [15,16]; however, such conventional approaches exhibit limitations in handling long-range temporal dependencies for time-series-based displacement prediction.

Deep-learning models have further improved the performance of landslide displacement prediction. LSTM and BiLSTM represent mainstream models for landslide time-series forecasting [17,18,19]. A substantial body of relevant studies have been carried out for landslides in the Three Gorges Reservoir Area. Yang et al. applied LSTM to landslide displacement prediction for this region and verified its strengths in capturing temporal dependencies within displacement sequences [20]. Jiang et al. adopted a displacement-component decomposition strategy and combined it with KNN for optimized input selection, which improved prediction accuracy [21]. Duan demonstrated that incorporating multivariate rainfall and reservoir water level data can boost the prediction performance of LSTM models [22]. For step-like landslides under small-sample conditions, Yu et al. proposed an enhanced training strategy to enable effective deep-learning with limited training samples [23].

Embedding attention mechanisms can further refine model prediction performance. Zhang et al. integrated time-frequency decomposition with gated recurrent units to achieve high-precision landslide prediction [24]. Yang et al. constructed an attention-based CNN-LSTM model for spatiotemporal displacement prediction of coupled landslides and obtained satisfactory accuracy [25].

Collectively, these existing approaches exhibit distinct strengths and inherent limitations when applied to reservoir-zone landslides within the TGRA. Statistical models deliver simple implementation but struggle to capture strong nonlinear deformation triggered by coupled rainfall and reservoir water level fluctuations. Traditional machine learning methods (e.g., SVR, random forest) achieve satisfactory performance for relatively stable deformation periods; nevertheless, they lack the capacity to model long-range temporal dependencies of sequential monitoring records. Standard LSTM and BiLSTM architectures are widely adopted for landslide time-series forecasting, yet most of them rely on single-point rain-gauge rainfall inputs and static feature concatenation, which cannot accommodate spatial rainfall heterogeneity and dynamically shifting dominant triggering factors during different creep stages. Recent attention-enhanced deep-learning models improve temporal feature extraction; however, they rarely design modality-aware dynamic fusion modules and seldom couple model outputs with physical creep-theory-driven multi-level warning frameworks specifically for TGRA reservoir landslides. These practical constraints motivate the technical improvements proposed in this manuscript.

It is worth noting that most existing deep-learning-based landslide-prediction studies focus on network-structure optimization. Less attention has been paid to input-oriented issues such as multi-sensor data preprocessing and spatial heterogeneity of rainfall. Conventional investigations mostly rely on observations from single rain-gauge stations and fail to capture the spatiotemporal distribution of mountainous precipitation. In the field of spatial interpolation, inverse distance weighting (IDW) and Kriging interpolation are commonly adopted to estimate rainfall distribution [26]. Nevertheless, ordinary Kriging relies on manually fitted variograms. This manual fitting process may introduce subjective bias. Particle swarm optimization supports global parameter search for interpolation and improves rainfall-estimation accuracy [27,28].

These technical bottlenecks have persisted for several practical reasons. First, high-resolution multi-source rainfall datasets integrating ground stations, meteorological radar, and multi-lead-time forecast products are not easily accessible for most landslide research groups. Second, fine-grained spatial rainfall reconstruction requires additional geospatial pre-processing pipelines and brings extra computational overhead. Third, many publicly available landslide monitoring datasets only provide single-point rain-gauge observations, which encourages researchers to prioritize neural-network structural optimization rather than input-oriented data enhancement. Driven by easy-to-obtain performance gains from network tuning, many studies mainly focus on model architecture improvements while overlooking input-data quality and adaptive multimodal fusion strategies. Against this background, three main research limitations are summarized as follows:

(1) Input-data-oriented bottlenecks for reservoir landslide modeling. On the one hand, rainfall inputs are often restricted to single-point rain-gauge measurements; such input schemes fail to capture spatial rainfall heterogeneity in mountainous TGRA terrain. On the other hand, existing multimodal integration mostly adopts static concatenation or fixed-weight fusion, which cannot adaptively adjust modal contributions under varying landslide creep stages. Both sub-problems degrade model performance for reservoir landslide prediction.
(2) Incomplete multi-temporal-scale prediction coverage. Existing research predominantly focuses on 1–3 d short-term displacement prediction. Integrated prediction frameworks supporting continuous short-to-medium-and-long-term horizons (1 d–15 d) are still scarce, which cannot meet differentiated engineering decision-making requirements.
(3) Absence of creep-theory-coupled multi-level early-warning mechanisms. Most models only output numerical displacement predictions, without mapping model outputs onto physical landslide creep evolution stages. Physics-guided multi-level warning systems are lacking, which impedes direct practical engineering decision-making support.

To address the above-mentioned drawbacks, this paper proposes a multi-scale landslide displacement prediction and multi-level early-warning framework based on multimodal data fusion for the Three Gorges Reservoir Area. The primary contributions of this paper, together with their responses to the corresponding research limitations, are presented below:

(1) Fine-grained rainfall reconstruction scheme of PSO–Kriging for reservoir-area multi-source sensing rainfall observations. High-accuracy rainfall estimation is realized with a grid resolution of 500 m. Short-term (0–6 h) rainfall prediction is achieved via fine-grained gridding of weather-radar echo images. Medium-and-long-term rainfall prediction (24 h, 48 h, 72 h, 96 h, 120 h, 144 h, and 168 h) is implemented by gridding precipitation-forecast products released by the China Meteorological Administration (CMA). Both strategies achieve a spatial resolution of 500 m. This contribution addresses the rainfall-spatial-representation sub-problem within Limitation (1).
(2) BiLSTM–Attention-based temporal-feature extraction. Two independent BiLSTM–Attention feature-extraction networks are constructed for displacement and reservoir water level modalities. The attention mechanism automatically learns importance weights for each time step and effectively captures both short-term and long-term dependencies within reservoir water level and displacement sequences. This component resolves the static-fusion sub-problem described in Limitation (1).
(3) Gated-attention mechanism for adaptive multimodal fusion. A gated-attention fusion module is designed to realize cross-modal dynamic weighting. It can adaptively match different landslide-deformation stages. During the constant-velocity creep stage, the model automatically reduces weights for the rainfall modality and increases weights for displacement-inertia features. During the accelerated-creep stage, weights assigned to rainfall and reservoir water level modalities rise, which effectively improves the accuracy and stability of the landslide-prediction model. This component further complements the adaptive multimodal fusion capability to tackle Limitation (1).
(4) Multi-scale prediction and multi-level early warning. A multi-scale displacement prediction framework ranging from 1 day to 15 days is established. Combined with the classical five-stage creep theory, a four-level landslide early-warning system is constructed. It provides multi-level decision-making support for managers, ranging from landslide emergency response to medium-and-long-term trend assessment. This work addresses the limitation of incomplete multi-temporal-scale prediction coverage formulated in Limitation (2), and addresses the absence of creep-theory-coupled multi-level early-warning mechanisms described in Limitation (3).

The remainder of this paper is organized as follows. Section 2 presents the theoretical foundations and methodology of the proposed framework, including five-stage landslide creep theory, fine-grained rainfall-gridding workflows, PSO–Kriging interpolation, BiLSTM–Attention temporal feature extraction, gated-attention multimodal fusion, and the dual-task prediction-warning pipeline. Section 3 describes experimental settings and analyzes experimental results, including dataset description, evaluation metrics, rainfall interpolation experiments, temporal-model comparison, ablation experiments for multimodal fusion, and multi-level early-warning results. Section 4 provides further discussion: we interpret ablation outcomes, gated-attention weight interpretability, and the effectiveness of fine-grained rainfall pre-processing. Section 5 summarizes major findings, analyzes existing limitations, and points out directions for future research.

## 2. Theoretical Foundations and Methodology

**Figure 1:**
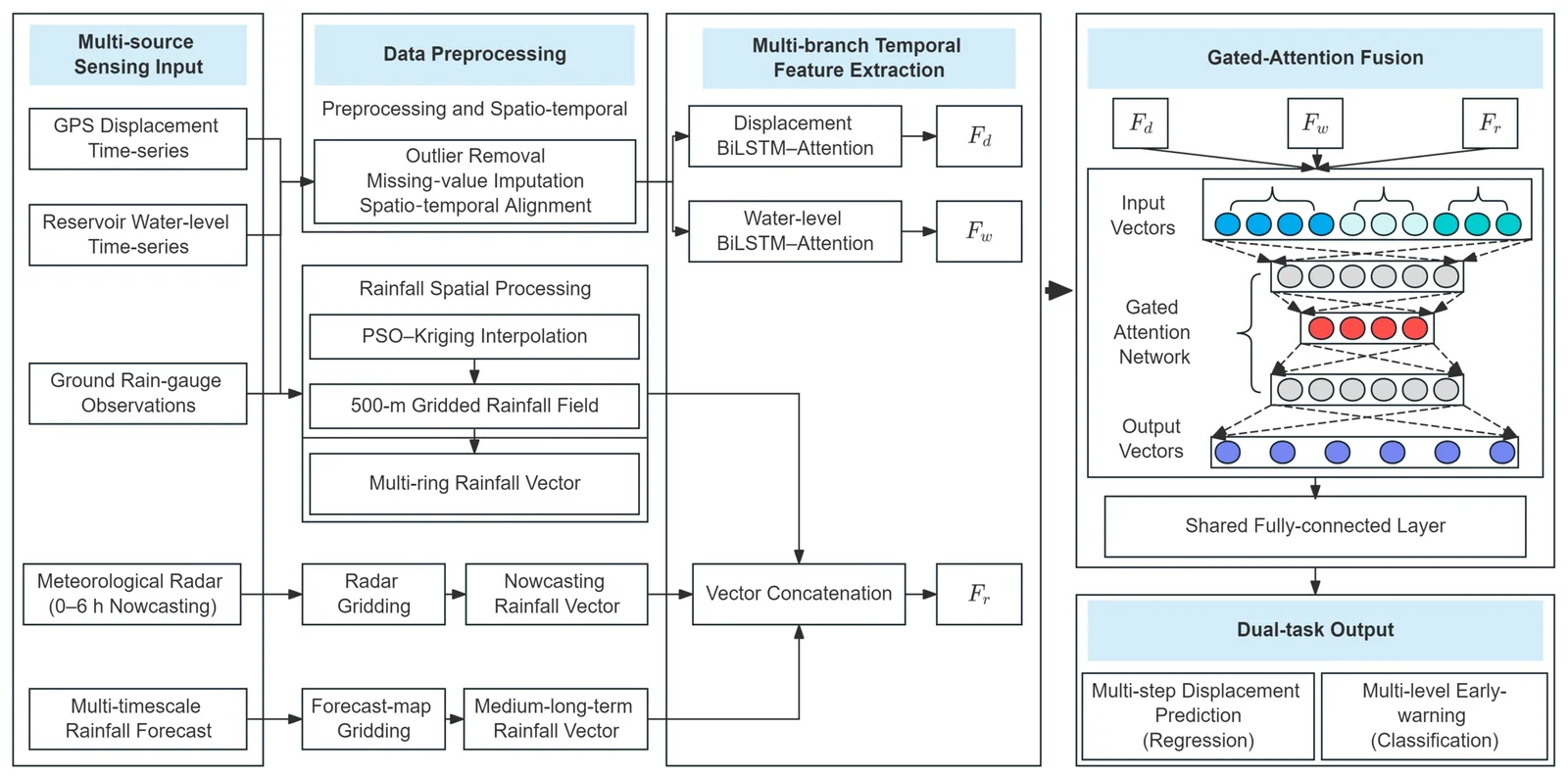
Overall technical framework for multi-scale landslide displacement prediction and multi-level early-warning based on multi-source sensing.

The overall system architecture of the proposed multi-scale landslide displacement prediction and multi-level early-warning framework is illustrated in Figure 1. This framework consists of five core components: multi-source sensing input, data preprocessing, multi-branch feature extraction, gated-attention fusion, and dual-task output.

GPS displacement, reservoir water-level, rain gauge, meteorological radar, and multi-timescale rainfall-forecast data serve as system inputs. Outlier removal, missing-value imputation, and spatiotemporal alignment are performed as preprocessing steps. PSO–Kriging interpolation is then implemented to generate a 500 m gridded rainfall field and multi-ring rainfall vectors. The short-term forecast rainfall vectors derived from radar products are concatenated with medium-and-long-term forecast rainfall vectors to obtain comprehensive rainfall features.

Two parallel BiLSTM–Attention branches are adopted to extract displacement features and water-level features. The gated-attention fusion network adaptively fuses multimodal features, which are further fed into a shared fully-connected layer. Finally, the dual-task output layer accomplishes multi-step displacement regression prediction and multi-class landslide early-warning classification. Subsequent subsections elaborate on the fundamental theories, algorithm principles, and implementation details for each module within this architecture.

### 2.1. Five-Stage Creep Theory of Landslides

Slope rock–soil mass deformation is driven by long-term combined effects of gravity, historical displacement inertia, rainfall, and reservoir water level fluctuation. The displacement-time (*S* − *t*) evolutionary law provides a core theoretical foundation for landslide monitoring, deformation prediction, and multi-level early warning.

**Figure 2:**
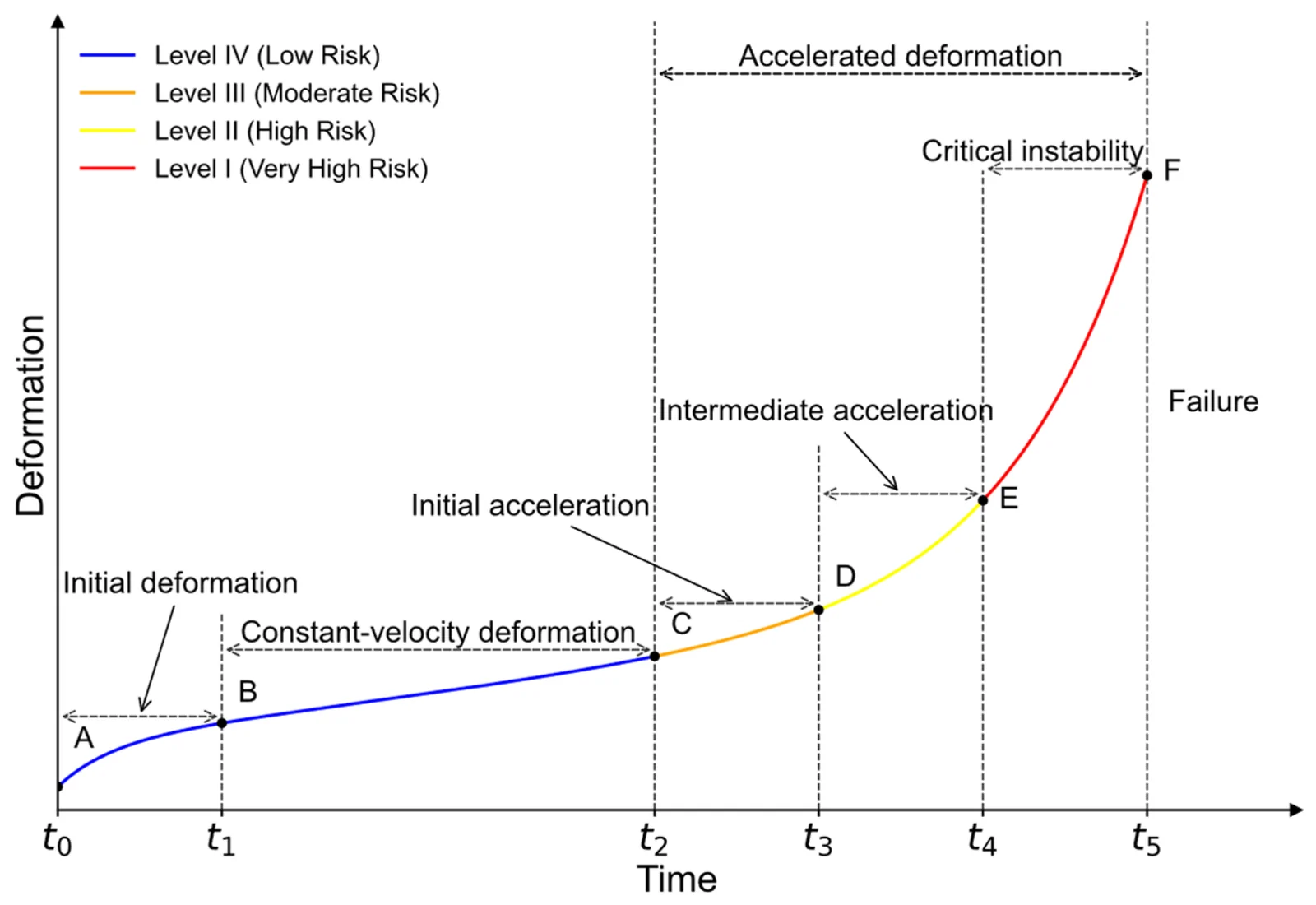
Evolution diagram of five-stage landslide deformation.

In 1969, Saito proposed the three-stage creep model for landslides. This model can only characterize the overall evolutionary trend of catastrophic sudden landslides. It cannot finely distinguish deformation rates and early-warning thresholds corresponding to different deformation stages [29]. Xu Qiang’s research team further improved and supplemented the five-stage landslide creep theory based on abundant field-measured landslide monitoring curves [30]. The standardized displacement-time evolutionary curve is shown in Figure 2.

This theory divides the whole landslide evolution process into five deformation stages defined by time nodes *t*_0_ to *t*_5_:

(1) Initial deformation stage (*t*_0_ − *t*_1_): The slope remains generally stable, while local damage accumulates continuously inside rock–soil masses.
(2) Constant-velocity deformation stage (*t*_1_ − *t*_2_): A through-going shear surface is formed. The deformation rate stays nearly constant, and displacement increases approximately linearly. This stage is vulnerable to rainfall and reservoir water level disturbances and represents the optimal window for landslide mitigation.
(3) Initial-acceleration deformation stage (*t*_2_ − *t*_3_): The landslide deformation rate rises slowly. Slope deformation becomes highly sensitive to external impacts, and higher monitoring frequency should be adopted at this stage.
(4) Accelerated deformation stage (*t*_3_ − *t*_4_): The curvature of the deformation curve keeps increasing, and the slope gradually approaches global instability with irreversible deformation trends. Intense rainfall nowcasting events can further accelerate slope failure.
(5) Failure stage (*t*_4_ − *t*_5_): The displacement rate grows exponentially. Immediate evacuation of residents in hazard-prone zones is mandatory.

In this work, the refined five-stage landslide-deformation theory is adopted. Deformation rates and tangent angles of deformation curves are used to distinguish multi-level deformation states. It provides theoretical support for manual labeling of early-warning samples in datasets and the subsequent construction of the four-level early-warning model.

### 2.2. Fine-Grained Gridding of Rainfall Data

Rainfall acts as the most direct external trigger for reservoir-zone landslides [31]. Existing studies indicate that landslide activities are closely associated with rainfall distributions within a 5 km radius around monitoring sites. Therefore, acquiring fine-grained rainfall data for target monitoring sites is critical for improving landslide-prediction accuracy.

Different strategies are adopted for rainfall prediction at distinct temporal scales, namely nowcasting rainfall (0–6 h) and medium-and-long-term rainfall (24 h–168 h). Radar echo maps are gridded at fine resolution to obtain rainfall information for target sites and surrounding areas for nowcasting rainfall. For medium-and-long-term rainfall, precipitation forecast maps released by the China Meteorological Administration are processed with fine-grained gridding to accurately extract rainfall values for target sites and their surroundings.

The proposed fine-grained rainfall-gridding method achieves a spatial grid resolution of 500 m × 500 m. It can be applied both to the reconstruction of historical rainfall fields and the prediction of future rainfall. For historical rainfall, measured data from hundreds of rain-gauge stations across the Three Gorges Reservoir Area are processed by the PSO–Kriging interpolation algorithm to reconstruct regional rainfall maps. Fine-grained gridding is further performed to obtain accurate rainfall values within each 500 m grid.

**Figure 3:**
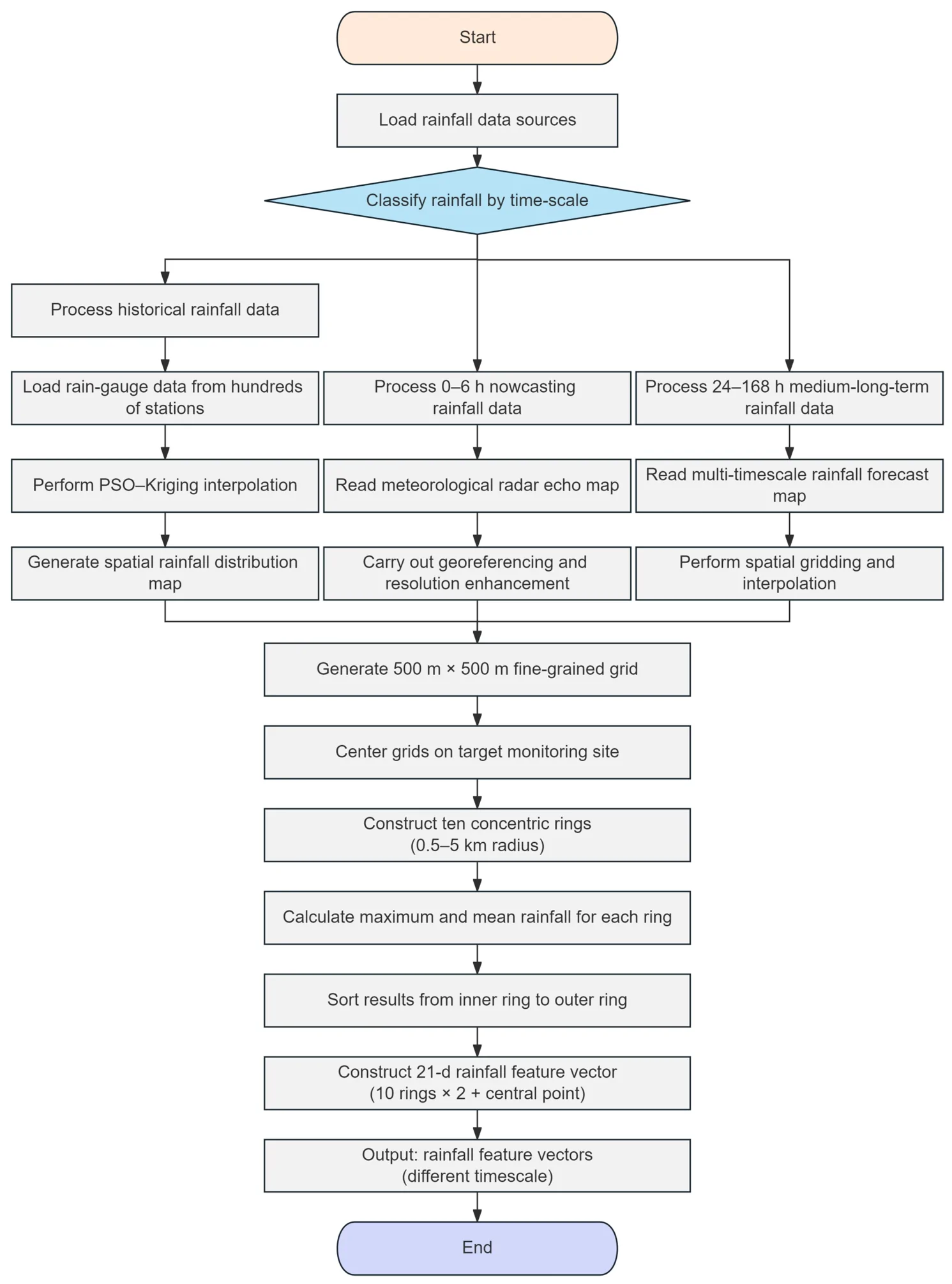
Flowchart of rainfall fine-grained gridding and rainfall feature vector extraction.

For the specific implementation of fine-grained gridding, rainfall maps are divided into dense grids. Centered at each target monitoring site, ten concentric rings are extended outward with a 500 m interval, covering a spatial range from 0.5 km to 5 km. The maximum and mean rainfall values are calculated for each ring. These statistics are arranged from the innermost ring to the outermost ring to form a 21-dimensional rainfall feature vector (10 rings × 2 statistical metrics + 1 central-site rainfall value). This vector characterizes rainfall information for the target monitoring site, as illustrated in Figure 3.

Fine-grained rainfall gridding consists of the following three components:

(1) Fine-grained gridding for historical rainfall: Fine-grained rainfall vectors around target sites are extracted to provide high-quality inputs for reservoir water level and landslide displacement prediction.
(2) Fine-grained gridding for future nowcasting rainfall (0–6 h): Fine-grained gridding is performed on meteorological radar echo maps to extract nowcasting rainfall feature vectors at a spatial resolution of 500 m.
(3) Fine-grained gridding for future medium-and-long-term rainfall (24–168 h): Multi-timescale rainfall forecast maps (24 h, 48 h, 72 h, 96 h, 120 h, 144 h, 168 h) released by the China Meteorological Administration are processed via fine-grained gridding. Rainfall feature vectors with 500 m resolution are then extracted.

### 2.3. Particle-Swarm-Optimization-Improved Kriging Interpolation Algorithm

Landslide occurrence is directly related to rainfall conditions at target monitoring sites and their surrounding areas [32]. Historical rainfall data are obtained from ground rain-gauge stations. Future rainfall data are derived from fine-grained gridding of meteorological radar echo maps and rainfall forecast maps. Restricted by deployment costs, rain-gauge stations in the reservoir area are sparsely distributed, which leads to low accuracy in characterizing spatial rainfall patterns. Therefore, this work adopts particle-swarm-optimized Kriging interpolation. It reconstructs continuous regional rainfall fields from discrete station rainfall observations to obtain high-accuracy spatial distribution of historical rainfall.

#### 2.3.1. Kriging Interpolation Algorithm

The Kriging algorithm takes full account of the spatial relative positions between measured sample points and interpolation points. It can realize the optimal unbiased estimation for regionalized variables [33]. The core calculation formula is shown in Equation (1).

(1) $$\left\{\begin{array}{c} \hat{Z}\left(u\right)=\sum_{i=1}^{n}\lambda_{i}Z(u_{i})\\ \sum_{i=1}^{n}\lambda_{i}=1 \end{array}\right.$$

where $\hat{Z}\left(u\right)$ is the predicted value at interpolation point *u*; *Z*(*u_i_*) denotes the measured value of the *i*-th sample point; *λ_i_* represents the weight of the *i*-th sample point; and *n* is the total number of sample points.

Interpolation weights are calculated via the variogram, as given in Equation (2).

(2) $$\left[\begin{array}{cccc} \gamma \left(x_{1}-x_{1}\right) & \cdots & \gamma \left(x_{1}-x_{n}\right) & 1\\ \vdots & \ddots & \vdots & \vdots \\ \gamma \left(x_{n}-x_{1}\right) & \cdots & \gamma \left(x_{n}-x_{n}\right) & 1\\ 1 & \cdots & 1 & 0 \end{array}\right]\times \left[\begin{array}{c} \lambda_{1}\\ \vdots \\ \lambda_{n}\\ \mu \end{array}\right]=\left[\begin{array}{c} \gamma \left(x_{1}-x_{0}\right)\\ \vdots \\ \gamma \left(x_{n}-x_{0}\right)\\ 1 \end{array}\right]$$

where *γ* is the variogram; *x*_0_ denotes the spatial position of the target prediction point; *μ* stands for the Lagrange multiplier.

The interpolation performance of the Kriging algorithm depends heavily on the selection of the variogram model and its key parameters (nugget *C*_0_, partial sill *C*, and range α). Traditional manual parameter tuning tends to introduce subjective bias and hardly yields a global optimal solution. Hence, the particle swarm optimization algorithm is introduced for parameter optimization in this study.

#### 2.3.2. Particle Swarm Optimization Algorithm

Particle swarm optimization (PSO) is a swarm-intelligence heuristic optimization algorithm [34]. It performs optimization by iteratively updating the position and velocity of each particle. The iteration rules are given in Equation (3).

(3) $$\left\{\begin{array}{c} V_{i}^{n+1}=\omega V_{i}^{n}+c_{1}r_{1}\left(P_{i}^{n}-X_{i}^{n}\right)+c_{2}r_{2}\left(P_{g}^{n}-X_{i}^{n}\right)\\ X_{i}^{n+1}=X_{i}^{n}+V_{i}^{n} \end{array}\right.$$

where $V_{i}^{n}$ and $X_{i}^{n}$ are the velocity and position of the *i*-th particle in the *n*-th iteration, $V_{i}^{n}=(v_{i1}^{n},v_{i2}^{n},\ldots ,v_{\mathrm{iD}}^{n})$ and $X_{i}^{n}=(x_{i1}^{n},x_{i2}^{n},\ldots ,x_{\mathrm{iD}}^{n})$ respectively. $P_{i}^{n}$ and $P_{g}^{n}$ denote the personal optimal position of the *i*-th particle and the global optimal position of the whole particle swarm at the *n*-th iteration. *ω* is the inertia-weight coefficient, which controls the retention degree of particle velocity. A larger inertia-weight coefficient strengthens global-searching capability, whereas a smaller value favors local-searching capability. *c*_1_ is the weight coefficient for accelerating particles toward the personal optimum. *c*_2_ is the weight coefficient for accelerating particles toward the global swarm optimum. *r*_1_ and *r*_2_ are random factors within the interval [0, 1] to guarantee stochastic-search performance.

**Figure 4:**
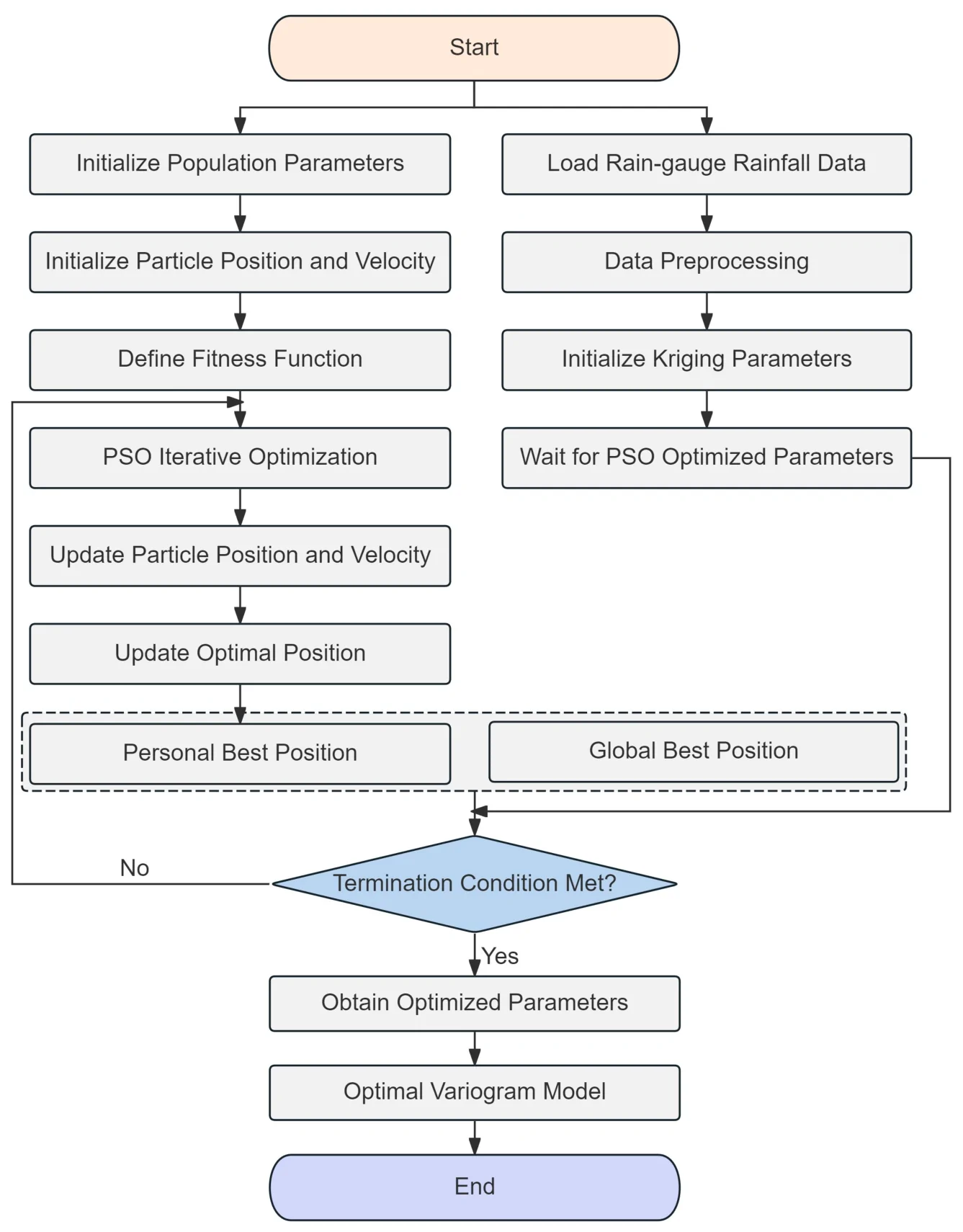
Flowchart of parameter optimization for PSO–Kriging interpolation.

In this study, the Gaussian model was selected as the variogram model. The particle swarm optimization algorithm was adopted to rapidly converge to the global optimal solution and obtain the optimal parameter combination. The workflow for parameter optimization is illustrated in Figure 4.

### 2.4. BiLSTM–Attention-Based Temporal-Feature Extraction

Long Short-Term Memory (LSTM) networks have been widely applied to time-series prediction tasks owing to their gating mechanism for handling long-range dependency problems [35]. Unidirectional LSTM only exploits historical temporal information. It exhibits severe response lags for mutation inflection points such as reservoir water-level regulation events and landslide-creep transitions.

Bidirectional Long Short-Term Memory (BiLSTM) integrates bidirectional temporal context. It is better suited for mining reservoir water level time series of reservoir-zone landslides. Nevertheless, it assigns identical weights to all time steps. Thus, it cannot highlight information from critical periods including reservoir regulation and accelerated landslide deformation [36].

**Figure 5:**
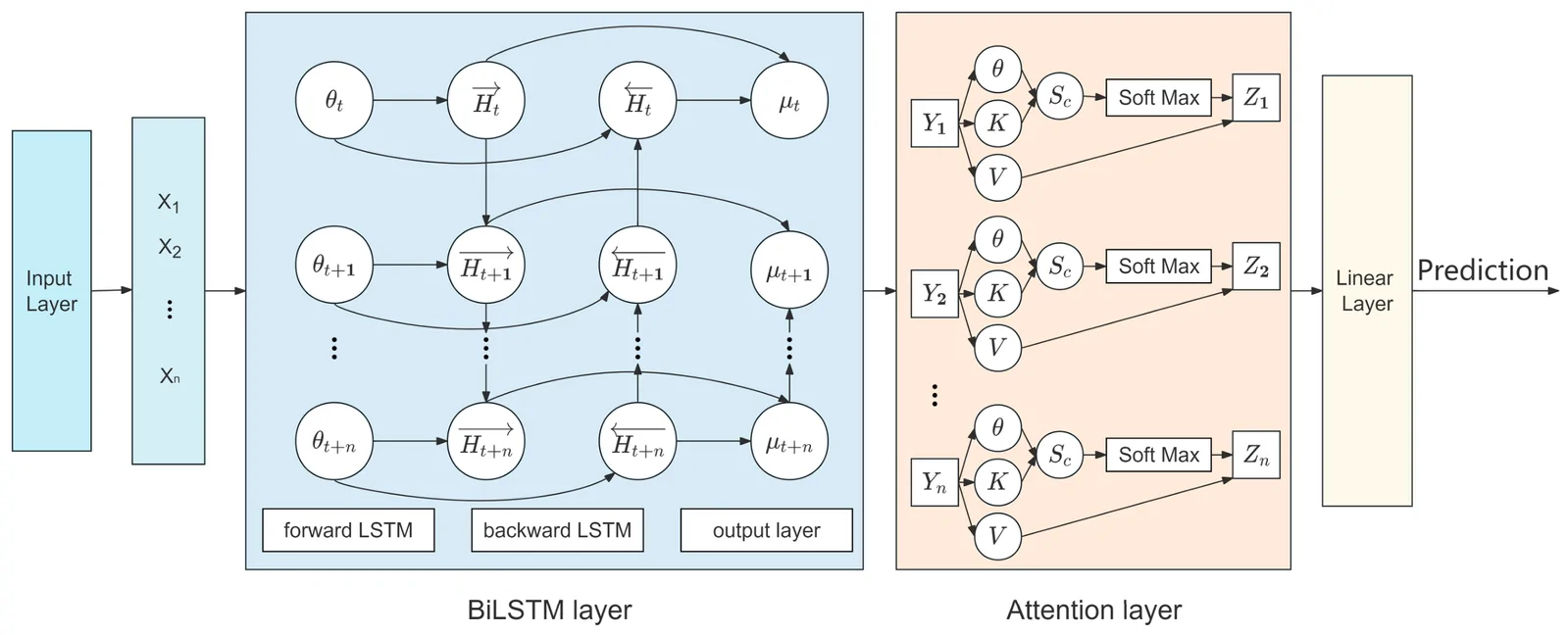
Network diagram of BiLSTM–Attention for temporal-feature extraction.

Therefore, an attention mechanism was introduced on the basis of BiLSTM, as shown in Figure 5. It adaptively adjusts weights for different time steps, strengthens feature contributions from critical deformation periods, and improves overall prediction accuracy.

Two independent BiLSTM–Attention networks with identical architectures were used for the displacement branch and reservoir water level branch. Each single-branch BiLSTM contains 64 units in its unidirectional hidden layer. The concatenated bidirectional hidden layer yields a 128-dimensional output. The model is trained iteratively on time-series datasets and finally outputs feature vectors. Among them, *F_d_* denotes the displacement-modal feature vector, and *F_w_* represents the reservoir water-level modal feature vector. Both vectors are 128-dimensional. The rainfall-modal features skip the BiLSTM–Attention network and are directly extracted from the fine-grained rainfall gridding workflow.

Model training hyper-parameters were set as follows. The Adam optimizer was adopted with an initial learning rate of 0.001. The dropout coefficient was set to 0.2 to mitigate overfitting for the temporal model. The batch size was set to 32. An early-stopping strategy was employed during model training.

### 2.5. Multimodal Feature Fusion Based on Gated-Attention Network

Landslide displacement evolution is jointly driven by multiple factors such as reservoir water level fluctuation and rainfall distribution at monitoring sites and surrounding areas [37]. The inputs of this study contain displacement and reservoir water level temporal features extracted by BiLSTM–Attention, as well as rainfall feature vectors concatenated from fine-grained feature vectors of historical rainfall, nowcasting rainfall and medium-and-long-term rainfall.

**Figure 6:**
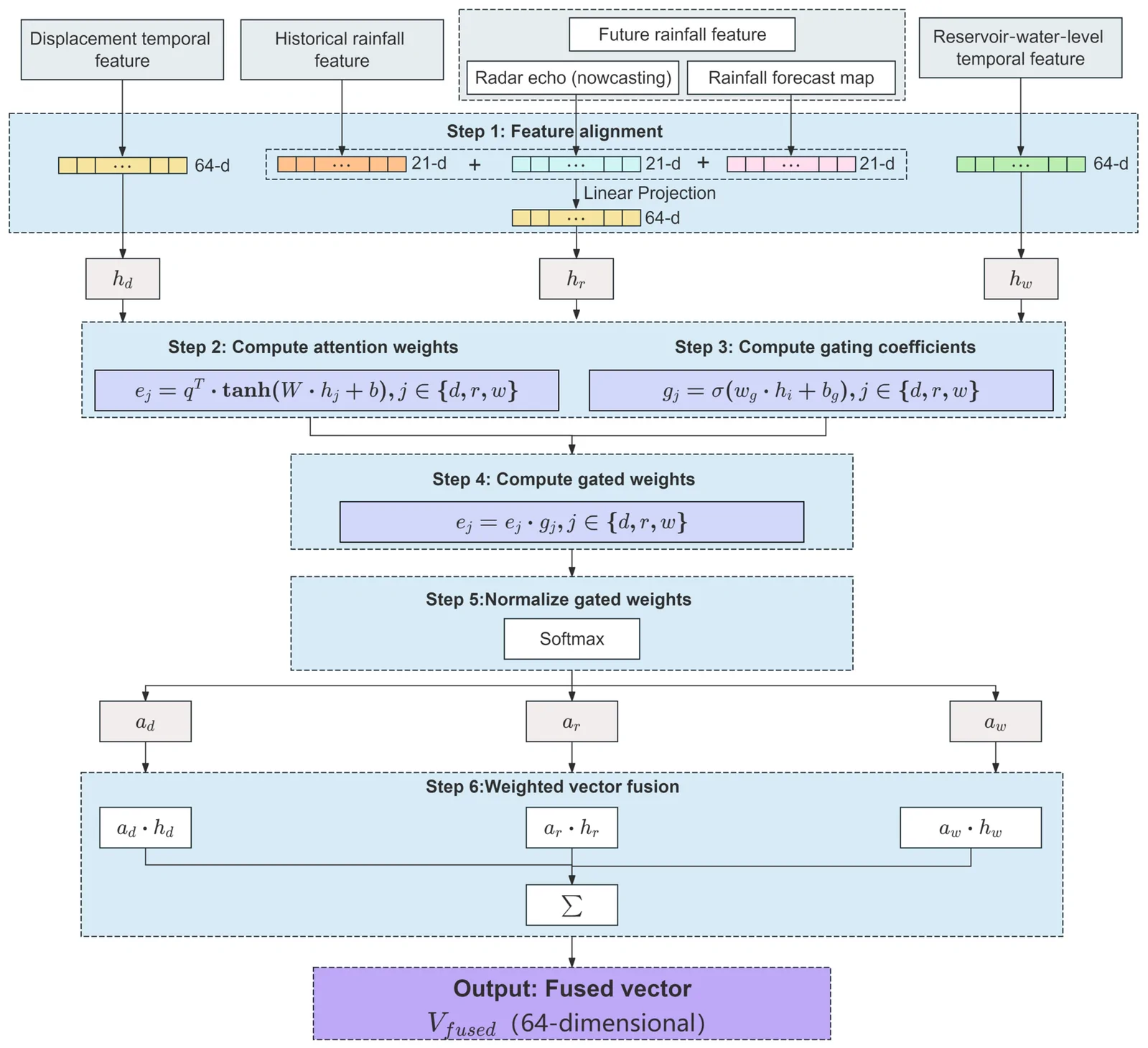
Calculation procedure of gated-attention-based multimodal feature fusion.

Complex coupling relationships exist among feature vectors of different modalities. Static fusion strategies fail to adapt to diverse creep stages of landslides [38]. To address this issue, we designed a gated-attention fusion layer, as shown in Figure 6. It realized dynamic weight assignment and adaptive fusion for deep multimodal features, so as to improve the prediction accuracy and generalization capability for landslides.

#### 2.5.1. Feature Vector Alignment

The historical landslide displacement time series and reservoir water level time series are fed into the BiLSTM–Attention model, which outputs 128-dimensional feature vectors *F_d_* and *F_w_*, respectively. Three separate 21-dimensional concentric-ring rainfall feature vectors are generated respectively from fine-grained gridding: (i) historical rainfall reconstructed by PSO–Kriging interpolation; (ii) 0–6 h nowcasting rainfall obtained from meteorological radar echo maps; (iii) 24–168 h multi-timescale medium-and-long-term rainfall from CMA forecast products. These three 21-dimensional vectors are directly concatenated to form the final 63-dimensional integrated rainfall feature vector *F_r_* (21 + 21 + 21 = 63).

Feature vectors of different modalities have inconsistent dimensions. Therefore, each modal feature is projected into a semantic space with a unified dimension for subsequent adaptive dynamic fusion. A dedicated independent linear layer was designed for each modality in this work. The corresponding calculations are given in Equations (4)–(6).

*h_d_* = *W_d_*·*F_d_* + *b_d_* (4)

*h_w_* = *W_w_*·*F_w_* + *b_w_* (5)

*h_r_* = *W_r_*·*F_r_* + *b_r_* (6)

where *W*_∗_ and *b*_∗_ denote learnable weight matrices, and *h*_∗_ ∈ *R^d^* represents intermediate hidden variables. *h_d_*, *h_w_*, and *h_r_* are feature vectors with identical dimensions after alignment for displacement-deformation, reservoir water level, and rainfall modalities, respectively. Three aligned feature vectors of equal dimension are obtained as outputs.

#### 2.5.2. Calculation of Attention Weights

The three aligned vectors are summed and fed into a feed-forward network together with a learnable context vector q to learn the weight of each feature vector. The calculation is shown in Equation (7).

*e_j_* = *q^T^*∙*tanh*(*W*∙*h_j_* + *b*),   *j* ∈ {*d*,*w*,*r*} (7)

where *W* denotes the weight matrix and *q* is the context vector. Both parameters can be obtained through iterative model training.

#### 2.5.3. Gating Coefficients

The Sigmoid function is adopted to generate gating coefficients ranging from 0 to 1. Attention weights are integrated with gating coefficients. This enables the gating mechanism to adaptively select or suppress certain features, as shown in Equations (8) and (9).

*g_j_* = *σ*(*W_g_*∙*h_j_* + *h_g_*),   *jϵ*{*d*,*w*,*r*} (8)

(9) $$\tilde{e_{j}}=e_{j}\cdot g_{j},\;\;\;j\epsilon \{d,w,r\}$$

The gating coefficient *g_j_* ∈ [0, 1] acts as a modality-wise soft switch: when *g_j_* approaches zero, the corresponding modal feature is heavily suppressed; when *g_j_* approaches one, the feature passes through with nearly full weight. By element-wise multiplication $\tilde{e_{j}}=e_{j}\cdot g_{j}$, the original attention score is modulated by a modality-specific gate, which distinguishes this gated-attention formulation from conventional vanilla attention. If the gating branch is removed (*g_j_* = 1 for all modalities), this formulation degenerates into standard multimodal attention fusion.

#### 2.5.4. Normalization of Attention Weights

The *Softmax* function is used to normalize attention weights into final fusion weights, which represent the importance of different landslide-related feature vectors, as given in Equation (10).

(10) $$\alpha_{j}=\frac{\exp(\tilde{e_{j}})}{\sum_{k\in \{d,w,r\}}\exp(e_{k})}$$

#### 2.5.5. Weighted Feature Fusion

The gated-attention mechanism is applied to achieve dynamic adaptive fusion of features from multi-source heterogeneous data. It enables subsequent landslide displacement prediction and multi-level early warning to extract valid features accurately according to variations in external environments. This contributes to improving model prediction accuracy and stability.

*V_fused_* = *α_d_*∙*h_d_* + *α_w_*∙*h_w_* + *α_r_*∙*h_r_* (11)

### 2.6. Landslide Displacement Prediction and Multi-Level Early Warning

After the fused multimodal feature vector is obtained from the gated-attention network, feature vectors extracted by the backbone network are fed into a shared linear layer. This study adopts a two-branch structure with shared backbone and independent task heads. It implements the regression task for multi-scale landslide displacement prediction and the classification task for landslide multi-level early warning.

The backbone network consists of BiLSTM–Attention branches for each modality, the rainfall-feature-vector extraction module, the gated-attention fusion network, and the shared fully-connected layer. The backbone network is shared by two tasks. Only the output-layer task heads are differentiated, which avoids reconstructing the complete network repeatedly.

**Figure 7:**
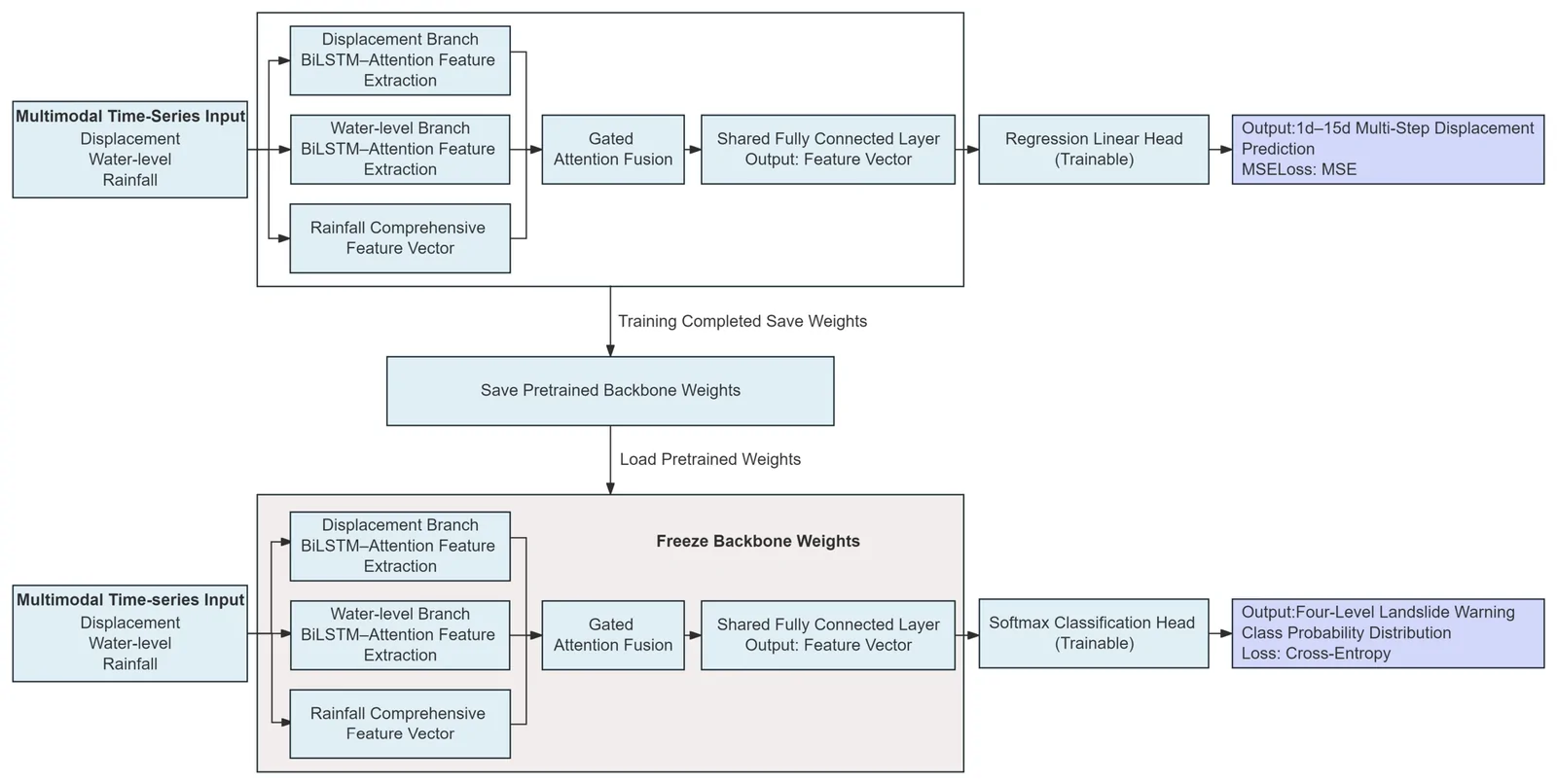
Schematic diagram of two-stage training network with frozen backbone for landslide displacement prediction and early-warning classification.

High-risk failure samples are limited in the landslide early-warning classification task. Noise introduced by small-sample classification may interfere with feature extraction of the backbone network. Therefore, a two-stage training strategy with frozen backbone was adopted for the two tasks. First, the backbone network was trained through the regression task of landslide displacement prediction. After training convergence, the backbone network was frozen. Only the task head was fine-tuned for the multi-level early-warning classification task. The network architecture is illustrated in Figure 7.

(1) Regression task for multi-scale landslide displacement prediction

Outputs from the shared fully-connected layer are connected to the linear head of the regression task. No activation function is configured for this head. Future landslide displacement values at multiple prediction horizons are directly output. The mean squared error loss function was adopted for the regression task to quantify the error between predicted displacement and field-measured monitoring displacement [39].

(2) Classification task for landslide multi-level early warning

This classification task reuses deep abstract features output by the shared fully-connected layer. Features are fed into a task head with the *Softmax* activation function to output probability distributions over four early-warning levels, so as to realize four-level landslide early-warning classification.

Based on the five-stage landslide creep theory, landslide evolution is divided into four warning levels: Level IV (low risk, initial/constant-velocity deformation), Level III (moderate risk, initial-acceleration deformation), Level II (high risk, accelerated deformation), and Level I (extremely high risk, failure stage). The cross-entropy loss function was used for this classification task.

## 3. Experimental Results and Analysis

### 3.1. Study Area and Dataset Description

The Three Gorges Reservoir Area (TGRA) is an important water-conservancy engineering region in the upper-middle reaches of the Yangtze River, China [40]. Periodic water-level regulation between 145 m and 175 m after reservoir impoundment alters seepage and stress conditions of bank slopes. It acts as a critical triggering factor for reservoir-bank landslides [41].

Multiyear monitoring datasets from six landslide sites in the TGRA were adopted in this study, including Baijiabao, Baishuihe, Bazimen, Shuping, Xintan, and Huangtupo. To conduct an out-of-site generalization test and strictly avoid data leakage, all samples from the Baijiabao landslide were completely excluded from model training and cross-validation. The training and validation datasets were constructed solely using data from the other five landslide sites (Baishuihe, Bazimen, Shuping, Xintan, Huangtupo).

Within these five non-test landslide sites, time-series samples were split into training and validation subsets via time-ordered partitioning without random shuffling, to prevent temporal data leakage. Five-fold cross-validation was performed only on this combined training–validation pool for hyperparameter tuning and ablation-experiment comparison. The Baijiabao dataset was never accessed during model training, cross-validation or hyper-parameter optimization and served purely as the out-of-site independent test set for final performance evaluation. After sliding-window sample generation, the training set contained 19,714 samples, the validation set contained 4929 samples, and the Baijiabao independent test set contained 2463 samples.

The dataset mainly contains time-series observations of landslide displacement, reservoir water level, and rainfall. The data were obtained from the National Cryosphere Desert Data Center of China (https://www.ncdc.ac.cn/). The time span of the dataset ranges from 2007 to 2024, covering approximately 6200 days with around 27,000 monitoring records in total.

Weather-radar echo maps are utilized to acquire nowcasting rainfall information. Multi-lead-time future rainfall forecast maps (24 h, 48 h, 72 h, 96 h, 120 h, 144 h, 168 h) are employed to obtain medium-and-long-term rainfall information. The above-mentioned meteorological image data are sourced from the National Meteorological Center of China (https://nmc.cn/).

**Figure 8:**
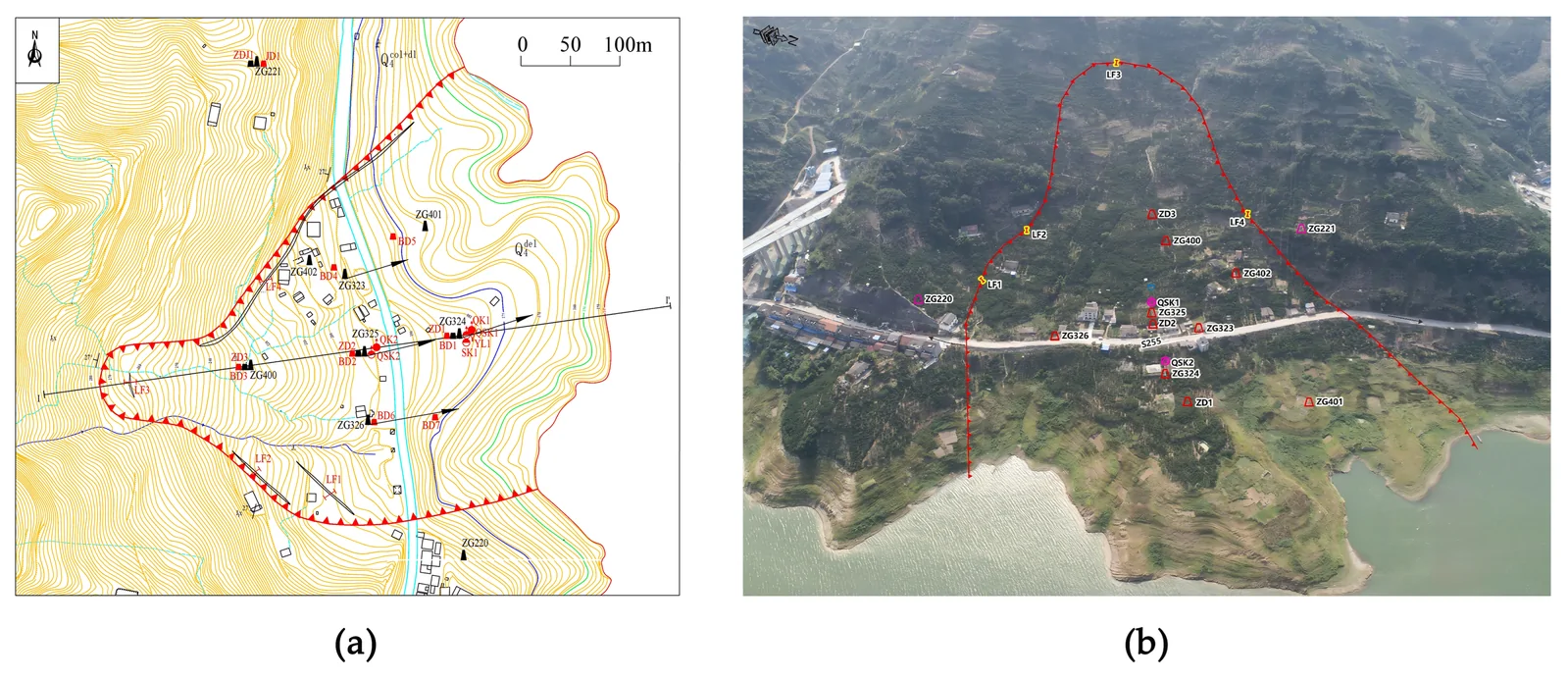
Overview of the Baijiabao landslide and distribution of surface monitoring points in the Three Gorges Reservoir Area. (**a**) Layout of landslide monitoring points. (**b**) Remote-sensing overview of the landslide.

**Figure 9:**
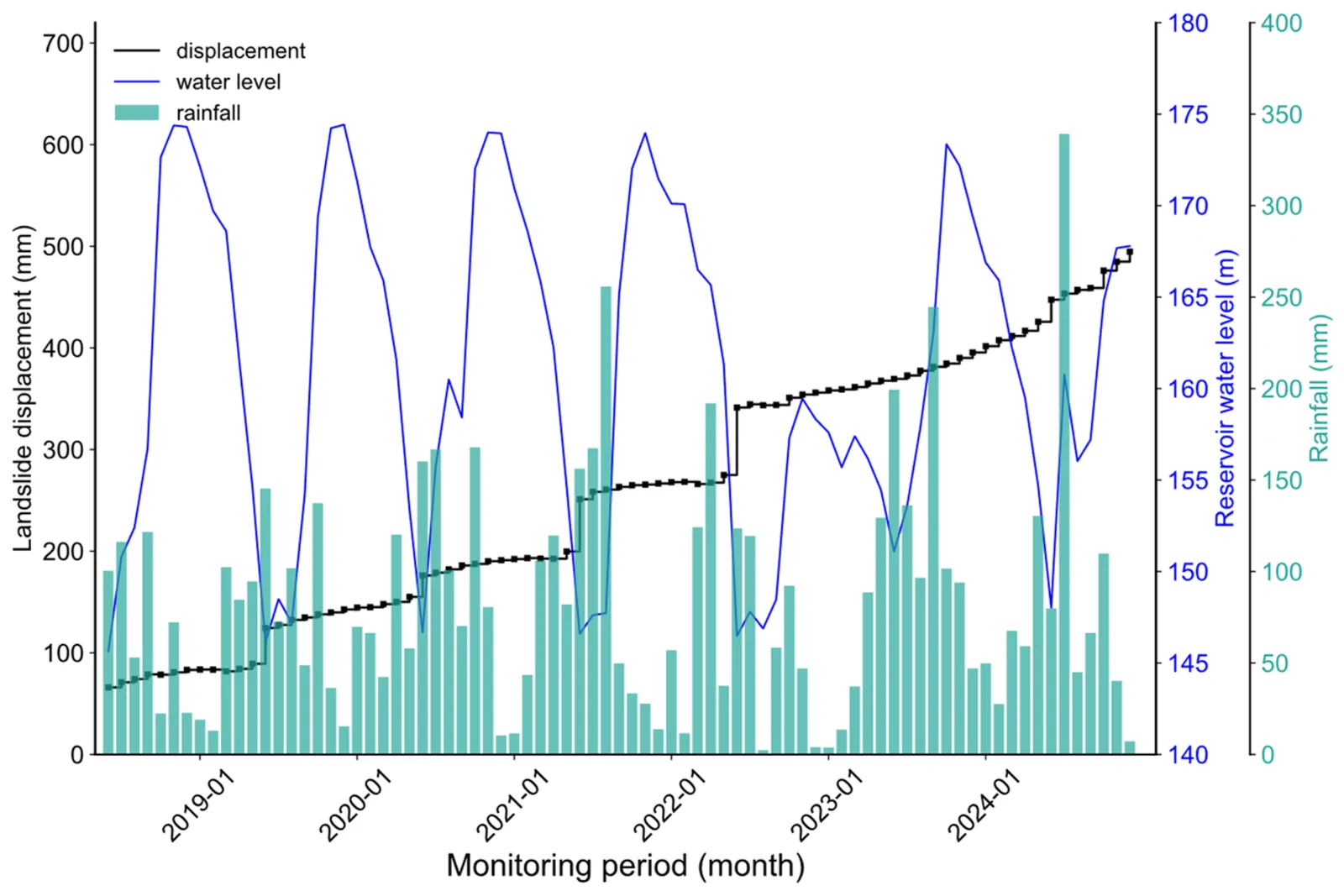
Temporal evolution curves of displacement, reservoir water level, and rainfall for the Baijiabao landslide in the Three Gorges Reservoir Area.

Taking the Baijiabao landslide, a typical landslide case in the Three Gorges Reservoir Area, as an example, Figure 8 illustrates the layout of monitoring points and the remote-sensing overview of this landslide. Furthermore, Figure 9 visualizes the monitoring data of the Baijiabao landslide from 2018 to 2024. It reveals the variation patterns of landslide displacement, reservoir water level, and rainfall.

The reservoir water level exhibits obvious periodic variations. Its peak and valley values show a certain lag effect relative to rainfall. Landslide displacement is positively correlated with rainfall and negatively correlated with reservoir water level. The landslide displacement presents typical multi-stage evolutionary characteristics. Its variation trend is basically consistent with the five-stage deformation evolution law shown in Figure 2.

### 3.2. Evaluation Metrics

#### 3.2.1. Regression Metrics

The rainfall prediction based on the PSO–Kriging interpolation algorithm, landslide displacement prediction, and reservoir water level prediction are all regression tasks. Three statistical metrics, namely mean error (ME), mean absolute error (MAE), and root-mean-square error (RMSE), are adopted to evaluate the prediction performance of the model [42].

Mean error reflects the systematic deviation direction of overall model predictions. Positive and negative errors counteract each other. Thus, this metric cannot characterize the magnitude of prediction error. It is applied to judge whether the model produces obvious under-estimation or over-estimation.

(12) $$\mathrm{ME}=\frac{1}{n}\sum_{i=1}^{n}\left(P_{a,i}-P_{e,i}\right)$$

Mean absolute error represents the average magnitude of numerical prediction error. Smaller MAE values correspond to higher model prediction accuracy.

(13) $$\mathrm{MAE}=\frac{1}{n}\sum_{i=1}^{n}\left|P_{a,i}-P_{e,i}\right|$$

Root-mean-square error amplifies individual prediction errors. It can effectively reflect the dispersion degree of prediction errors and the sensitivity to spatial variation.

(14) $$\mathrm{RMSE}=\sqrt{\frac{1}{n}\sum_{i=1}^{n}{\left(P_{a,i}-P_{e,i}\right)}^{2}}$$

where *P_a_*_,*i*_ is the predicted value of the *i*-th sample, *P_e_*_,*i*_ denotes the measured value of the *i*-th sample, and *n* is the total number of samples.

#### 3.2.2. Classification Metrics

Precision, Recall, and F1-score are utilized to assess the performance of the landslide multi-level early-warning task [42]. All metrics are calculated in the macro-average mode. Scores are computed for each early-warning category firstly. Arithmetic averaging is then performed across four warning levels. This treatment guarantees equal weight for every warning grade, as given in Equations (15)–(17).

(15) $$\mathrm{Precision}=\frac{\mathrm{TP}}{\mathrm{TP}+\mathrm{FP}}$$

(16) $$\mathrm{Recall}=\frac{\mathrm{TP}}{\mathrm{TP}+\mathrm{FN}}$$

(17) $$F1=2\times \frac{\mathrm{Precision}\times \mathrm{Recall}}{\mathrm{Precision}+\mathrm{Recall}}$$

where TP, FP, and FN are true positive, false positive, and false negative, respectively.

### 3.3. Dual-Source Rainfall Prediction

#### 3.3.1. PSO–Kriging Interpolation Algorithm

Rain-gauge stations in the Three Gorges Reservoir Area are spatially scattered. They only provide coarse-grained point-scale rainfall data, which cannot meet the requirements for fine-grained rainfall input of the multi-scale landslide deformation prediction model. Kriging interpolation can generate gridded rainfall fields across the whole region from station-observed rainfall records. Its interpolation accuracy directly determines the prediction performance of subsequent reservoir water level and landslide displacement prediction. In this work, interpolation performance was validated using rainfall datasets from 583 observation stations within the Three Gorges Reservoir Area.

Raw observations from rain-gauge stations provide coarse-grained rainfall information. The fine-grained rainfall field generated by interpolation exerts a significant influence on the accuracy of landslide-related prediction. Mean error, mean absolute error, and root-mean-square error are selected as evaluation metrics for interpolation. Five-fold cross-validation is performed to compare multiple interpolation algorithms. The experimental results are listed in Table 1.

**Figure 10:**
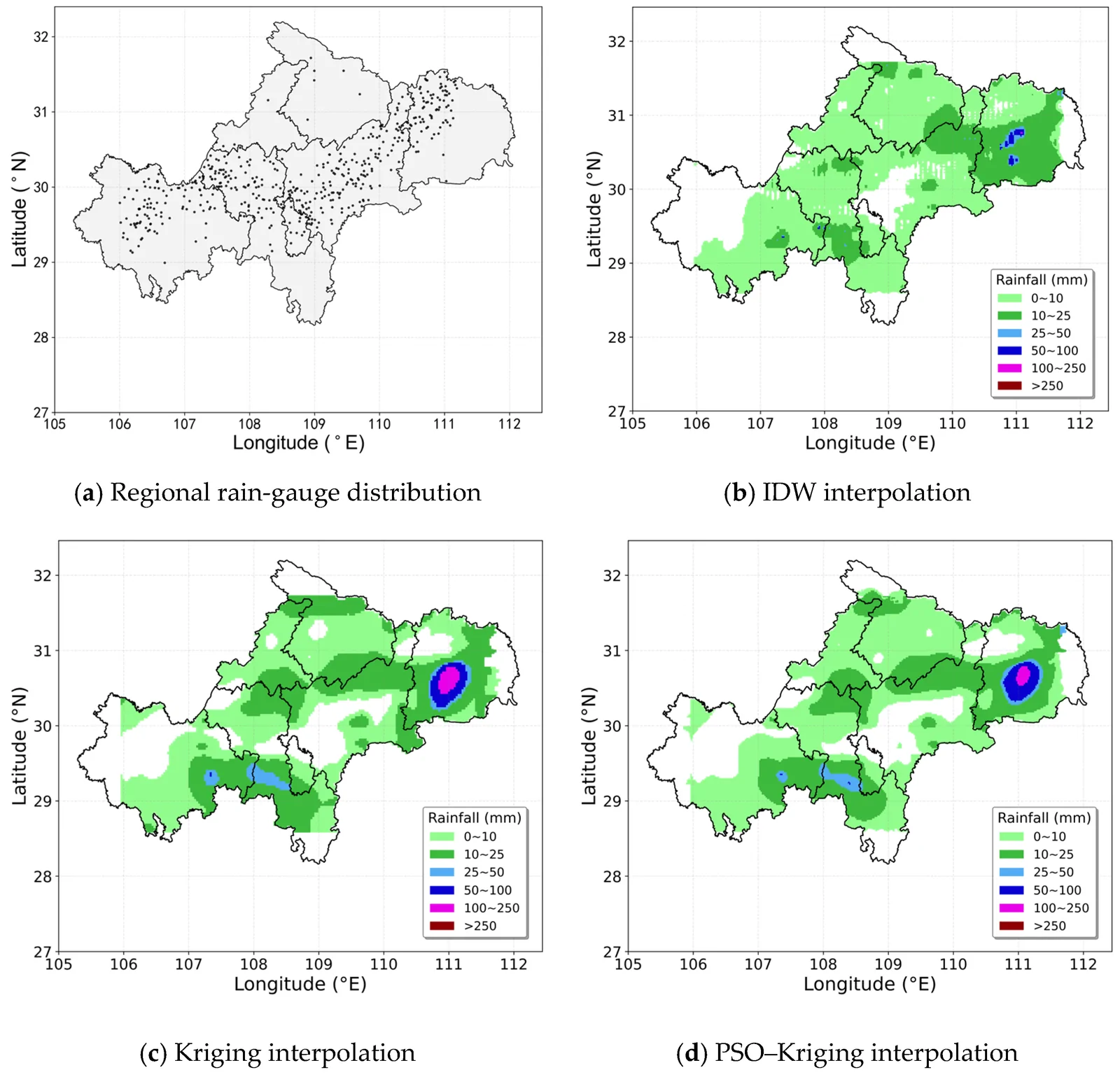
Comparison of rainfall interpolation results obtained by different algorithms for rain-gauge data in the Three Gorges Reservoir Area.

Table 1 presents quantitative error results of three interpolation algorithms obtained via five-fold cross-validation. IDW yields the highest errors among all metrics and exhibits obvious large local deviations. Combined with Figure 10b, the IDW interpolation result shows prominent stripe-like distortion and patch artifacts.

Conventional Kriging achieves higher accuracy than IDW. Nevertheless, manually fitted variograms hardly yield optimal parameters, and the interpolation output suffers from over-smoothing. As shown in Figure 10c, local heavy-rainfall signals are attenuated, which leads to the underestimation of rainfall extreme values.

The proposed PSO–Kriging achieves the best interpolation performance. Its mean error from five-fold cross-validation is only 0.49 ± 0.13 mm. Compared with conventional Kriging, MAE decreases by 21.8%, and RMSE decreases by 42.9%. The overall spatial patterns of rainfall reconstructed by Figure 10c,d are similar. However, PSO–Kriging can better reproduce the coverage of local heavy-rainfall centers and amplitude variation of rainfall intensity. It effectively alleviates the peak-clipping effect. The interpolated results are more consistent with the actual spatial distribution of rainfall in the TGRA. The output regional fine-grained rainfall vectors can provide reliable rainfall inputs for subsequent landslide prediction.

Manual variogram fitting produces a relatively large nugget value, as shown in Table 2. It overestimates random-noise components in rainfall fields and results in over-smoothed interpolation surfaces. Consequently, extreme values of local heavy rainfall are attenuated.

Global optimization via PSO yields a smaller nugget value, together with larger partial sill and range. These parameters better match the spatial statistical characteristics of rainfall in the TGRA. All error metrics are significantly improved. This indicates that parameter optimization serves as the key factor for accuracy enhancement of PSO–Kriging.

**Figure 11:**
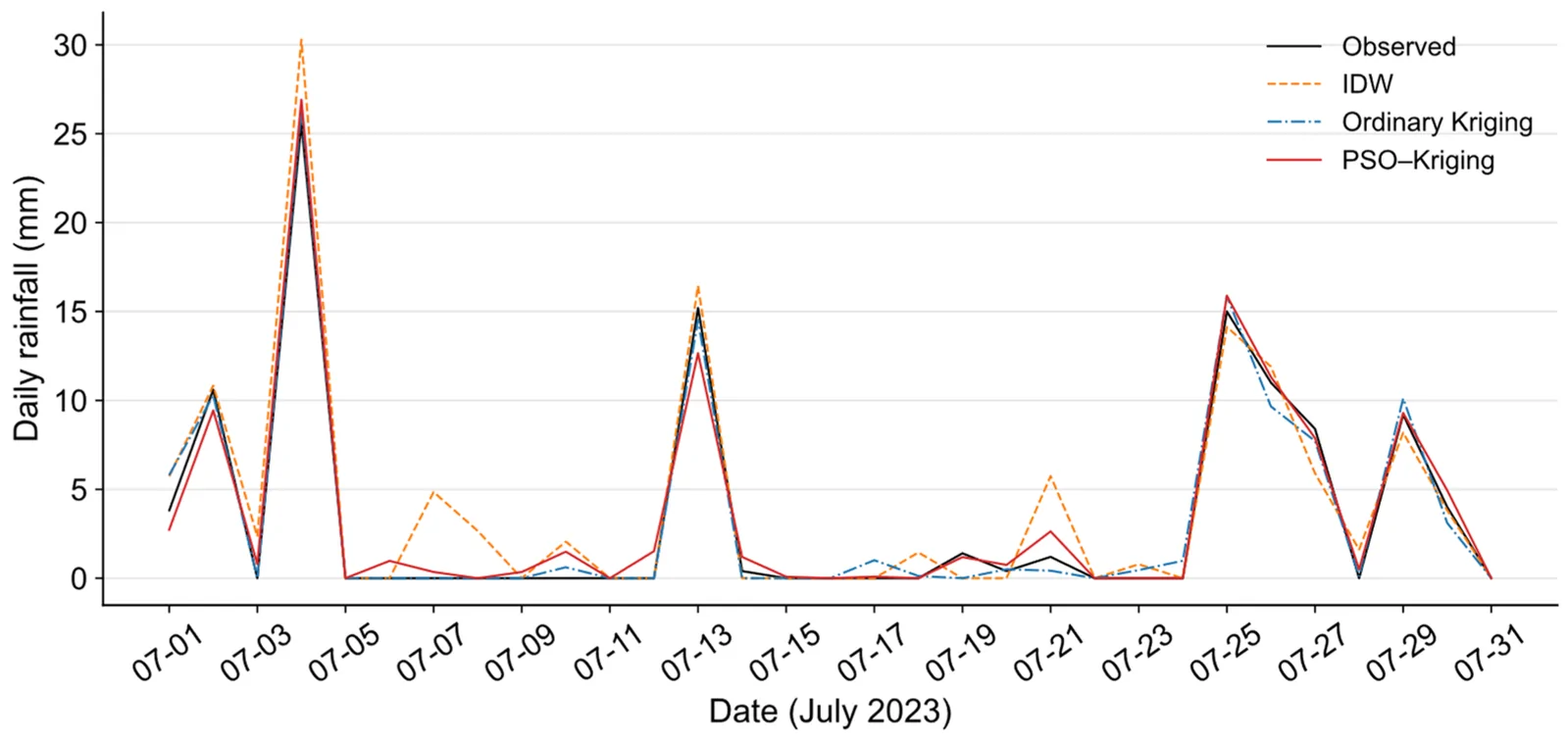
Comparison curves of daily rainfall time series at Baijiabao landslide rainfall station in the Three Gorges Reservoir Area (July 2023).

As shown in Figure 11, the rainfall time-series comparison curves in July 2023 from the rain-gauge station of the Baijiabao landslide include measured rainfall and fitted results of three interpolation algorithms. They further verify interpolation performance from the temporal perspective.

IDW relies solely on distance weights. Weights in regions near rain-gauge stations are over-amplified. It cannot adapt to complex mountainous terrain and suffers severe peak distortion. Limited by manually selected parameters, conventional Kriging tends to underestimate extreme rainfall events. PSO–Kriging captures both global trends and local abrupt rainfall variations. It achieves the best temporal fitting accuracy and stability and is well suited for complex terrain in the TGRA.

#### 3.3.2. Fine-Grained Gridding of Rainfall Distribution Maps

Fine-grained gridding of rainfall distribution maps covers historical rainfall and future rainfall. Historical rainfall originates from measured observations of hundreds of rain-gauge stations in the TGRA. The PSO–Kriging method described in Section 3.3.1 is first applied to reconstruct rainfall fields, followed by fine-grained gridding. For future rainfall, fine-grained gridding was directly conducted on weather-radar echo maps (nowcasting rainfall) and meteorological rainfall forecast maps (medium-and-long-term rainfall).

This work takes the nowcasting-rainfall weather-radar echo map of the TGRA as an example to illustrate the fine-grained gridding procedure. The weather-radar echo map has a dimension of 4567 × 3314 pixels. Its geographic extent ranges from 105° E to 112° E in longitude and 27° N to 32° N in latitude. After gridding with 3 × 3 pixel units, the spatial resolution of rainfall grids reaches approximately 444 m along the latitude direction and 500 m along the longitude direction.

**Figure 12:**
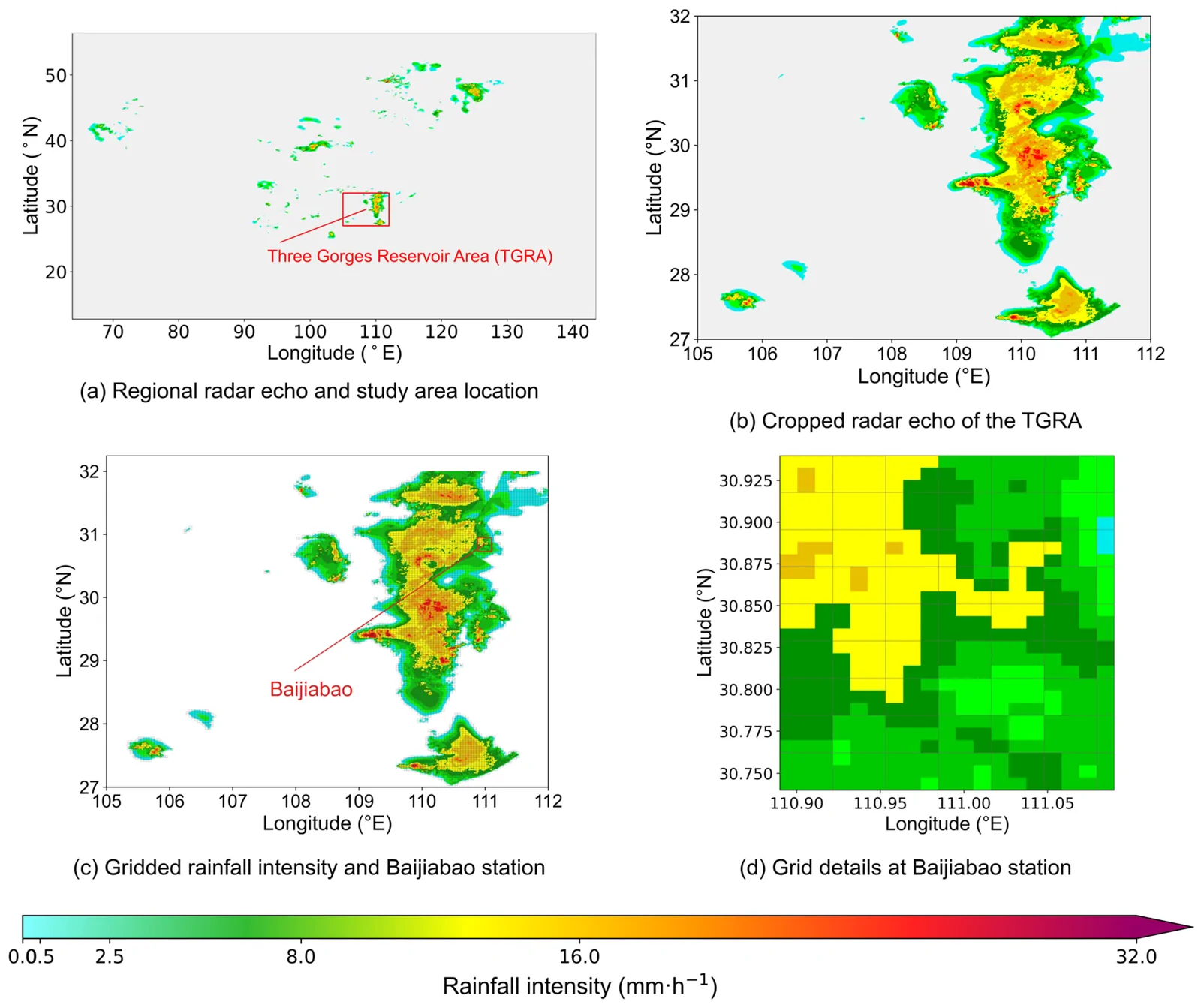
Fine-grained gridding processing of meteorological radar echo.

As shown in Figure 12, the first step is to obtain weather-radar echo maps covering the whole of China (Figure 12a). The regional extent of the TGRA is then cropped according to longitude–latitude coordinates (Figure 12b). In the second step, fine-grained gridding is performed on the cropped radar echo map of the TGRA. The gridding granularity is selected to guarantee a spatial rainfall resolution of approximately 500 m (Figure 12c). The third step extracts fine-grained grids covering the target monitoring site and its surrounding area (Figure 12d).

For rainfall-feature-vector construction, ten concentric rings are divided around the target monitoring site. The maximum rainfall and average rainfall are calculated for each ring. These statistics are arranged from the inner ring outward. Finally, a 21-dimensional rainfall feature vector is formed (10 rings × 2 statistical indicators + one central-site rainfall value).

To quantify the intrinsic skill of the input meteorological forecast products and disentangle landslide-model performance from meteorological-forecast errors, multi-lead-time CMA rainfall forecast outputs (24 h, 48 h, 72 h, 96 h, 120 h, 144 h, 168 h) were validated against ground-truth observations from 583 rain-gauge stations across the TGRA. The cross-validation metrics including ME, MAE, and RMSE for each forecast lead time are summarized in Supplementary Table S1. As expected, rainfall forecast errors gradually increase with longer lead times. These inherent meteorological forecast uncertainties will propagate into the 7 d and 15 d landslide displacement prediction outputs, which should be distinguished from the algorithmic error of our landslide prediction model.

### 3.4. Single-Modal Temporal Feature Extraction

#### 3.4.1. Dataset Partition Using Sliding-Window Strategy

Multi-year monitoring datasets from multiple landslide sites in the TGRA are adopted in this study, including Baijiabao, Baishuihe, Bazimen, Shuping, Xintan, and Huangtupo. The monitoring records span from 2007 to 2024, covering approximately 6200 days with around 27,000 monitoring entries. As illustrated in Figure 9, rainfall, reservoir water level, and landslide displacement exhibit obvious periodic patterns.

To make full use of periodic characteristics and expand the number of valid samples, a sliding-window strategy is employed for dataset partitioning. To investigate the influence of historical sequence length on model performance, five look-back window sizes were set: 30 days, 60 days, 90 days, 180 days, and 365 days. Comparative experiments were carried out based on the BiLSTM–Attention model. The five-fold cross-validation results are listed in Table 3.

The experimental results are shown in Table 3. The overall model accuracy gradually increases as the sliding-window size rises from 30 d to 90 d. Further enlargement of the window leads to accuracy degradation. Comprehensive comparison indicates that 90 d is the optimal look-back sliding window for this dataset.

#### 3.4.2. Training of the BiLSTM–Attention Model

As illustrated by the monitoring time-series visualization of the typical Baijiabao observation site in Figure 9, reservoir water level, landslide displacement, and rainfall show distinct variation characteristics. The time series of reservoir water level and landslide displacement possess strong continuity. Rainfall exhibits annual periodicity, while daily or multi-day rainfall events feature high randomness.

Therefore, independent BiLSTM–Attention networks are constructed to extract features only for reservoir water level and landslide displacement time series. Rainfall information is not fed into the temporal network. Instead, static feature vectors obtained from fine-grained gridding are directly imported into the model.

Datasets are built based on the optimal 90 d sliding window. Multi-scale predictions are performed for horizons of 1 d, 2 d, 3 d, 7 d, and 15 d. Comparative results of several widely-used temporal models including LSTM, BiLSTM, BiLSTM–Attention, and Transformer are summarized in Table 4 and Table 5. To ensure rigorous and fair comparison, all baseline models adopted identical input feature sets, 90-day look-back sliding window, Adam optimizer with an initial learning rate of 0.001, dropout = 0.2, batch size = 32, and the same early-stopping strategy (patience = 15). The architectural hyper-parameters, trainable parameters, and computational complexity in terms of floating-point operations (FLOPs) are reported in Supplementary Table S2. Runtime metrics for both training and inference were also reported for each model. All runtime statistics were measured on an NVIDIA RTX 3090 GPU. Transformer is a well-known deep-learning architecture relying on multi-head self-attention mechanisms, which can capture long-range temporal dependencies without recurrent units. It is selected here as a state-of-the-art time-series forecasting baseline for performance comparison against our BiLSTM–Attention backbone. As shown in Supplementary Table S2, Transformer possesses substantially more trainable parameters and higher computational overhead. Under our relatively limited landslide time-series sample size, the larger parameter space makes Transformer more susceptible to over-fitting risk.

It can be observed from Table 4 that BiLSTM–Attention outperforms LSTM and BiLSTM for reservoir water level prediction across all prediction horizons. It shows mixed performance compared with Transformer. For the 1 d horizon, the MAE of BiLSTM–Attention reaches 0.84 ± 0.31 m, and the model can accurately capture the variation trend of reservoir water level.

The overall prediction error accumulates and increases with longer prediction horizons. Under 7–15 d prediction scenarios, Transformer achieves slightly better performance on partial metrics. Nevertheless, BiLSTM–Attention exhibits more stable performance in terms of RMSE.

Results in Table 5 reveal that ME values remain small for short-term (1–3 d) landslide displacement prediction. ME increases moderately for medium-and-long-term (7–15 d) prediction. Such limited growth indicates no severe systematic bias in the model.

For short-term prediction, ME is close to zero, while RMSE is notably larger than MAE. This phenomenon suggests the existence of a small number of large-error samples. Prediction errors rise as the prediction horizon increases. BiLSTM–Attention achieves overall better performance than LSTM and BiLSTM.

In summary, BiLSTM–Attention exhibits stable and competitive prediction performance across all time scales for both reservoir water-level and displacement prediction tasks. Therefore, 1 d and 7 d are selected as representative horizons for short-term and medium-and-long-term early-warning in subsequent ablation experiments and multi-level early-warning tasks.

### 3.5. Multimodal Fusion with Gated-Attention Mechanism

To verify the effectiveness of gated-attention multimodal feature fusion for landslide prediction, six groups of ablation experiments were designed:

(1) Single-modal: Only historical displacement time-series are adopted as input. Reservoir water level and rainfall data are excluded.
(2) Partial-modal fusion: Fusion is performed between historical displacement series and reservoir water level information, or between historical displacement series and rainfall data.
(3) Direct concatenation: Multimodal features are directly concatenated without any attention mechanism.
(4) Fixed-weight fusion: The optimal weighting strategy is obtained via grid search for weighted fusion with fixed weights.
(5) Without gating unit: Only standard attention-weighted fusion is adopted, and the gating unit is removed.
(6) Proposed method: On the basis of the multimodal feature extraction pipeline in this work, gated attention is applied to realize adaptive fusion of features from different modalities.

Training sets, test sets, and all hyper-parameters are kept identical across ablation groups. Two representative prediction horizons, 1 d and 7 d, are used for ablation tests. The 1 d horizon represents short-term early-warning, whereas the 7 d horizon stands for medium-and-long-term early-warning. The ablation results are presented in Table 6.

As can be seen from Table 6, the complete proposed model (Ours) achieves the lowest ME, MAE, and RMSE at both 1 d and 7 d prediction horizons. The model without the gating unit (No Gating) yields larger errors than the full model. The direct-concatenation scheme produces higher errors than attention-weighted fusion. Some of its metrics are even inferior to those obtained from single-modal input. The fixed-weight fusion outperforms direct concatenation, yet all its error metrics are higher than those of the proposed gated-attention fusion scheme.

### 3.6. Multi-Level Early Warning

Dataset manual annotation was carried out based on monitoring records of multiple landslides in the TGRA and the five-stage creep theory of landslides shown in Figure 2. Sample labels for four-level early warning were divided according to landslide displacement rate and deformation patterns of the *S* − *t* curve, referring to characteristics of the five-stage creep evolution.

Reproducible quantitative labeling criteria were established to construct the four-level warning dataset. The 7-day sliding-window-averaged displacement rate ${\bar{v}}_{\mathrm{win}7}$ is adopted as the primary quantitative indicator for label assignment. For each timestamp sample, ${\bar{v}}_{\mathrm{win}7}$ is computed as the average daily displacement change within the trailing 7-day window ending at that moment.

This 7-day window is selected after balancing noise suppression and early-warning sensitivity. A single-day (1 d) rate is vulnerable to isolated monitoring outliers and transient displacement spikes, which may misclassify stable slopes into high-risk grades. A short 3-day window still cannot fully filter short-term noise. In contrast, a longer 15-day window would excessively smooth early weak acceleration signals and cause delayed early-warning responses. The 7-day window strikes a good balance: it suppresses random short-term anomalies while preserving the capacity to capture the onset of initial acceleration; furthermore, this time scale is consistent with the 7 d medium–long-term prediction horizon adopted in this work.

The curvature of the S-t displacement-time curve serves as a secondary morphological reference. For ambiguous boundary samples falling close to threshold boundaries, cumulative rainfall intensity and reservoir water-level fluctuation trends are further incorporated to assist label determination. The complete quantitative labeling specifications are summarized in Table 7.

As summarized in Table 7, the five-stage creep evolutionary sequence is aggregated into four practical warning grades for reservoir landslide engineering early-warning applications. Specifically, both the initial-deformation stage and constant-velocity-deformation stage are grouped into Level IV (low risk). From an engineering emergency response perspective, these two creep stages correspond to generally stable slope conditions; neither triggers urgent hazard-mitigation operations or resident-evacuation requirements, which justifies merging these two theoretical creep stages into a unified low-risk warning level.

Critically, it should be clarified that the ground-truth warning labels used for classification do not represent the instantaneous deformation state at the current input time-step. Instead, each sample’s warning label corresponds to the future deformation warning state at the end of the corresponding prediction horizon (1 d or 7 d ahead). This labeling paradigm implements genuine forecast-oriented early warning, rather than retrospective recognition of the already-observed deformation stage.

For samples located near phase-transition boundaries, label determination integrates rainfall, reservoir water-level and variation trends of monitoring curves. This strategy reduces annotation ambiguity. A four-level early-warning dataset is constructed for model training, and the Baijiabao landslide is selected as the test set for validation.

Identical data preprocessing pipelines are adopted for both the training and test sets with a 90-day sliding window. According to the multi-scale experimental conclusions in Section 3.4, 1 d and 7 d are chosen as representative horizons for short-term and medium-and-long-term early-warning in multi-level early-warning experiments.

Precision, Recall, and F1-score are used to evaluate early-warning performance, and all metrics are reported in the macro-average mode. Reported results correspond to mean ± standard deviation obtained from five independent runs with different random seeds. The experimental outcomes are summarized in Table 8.

As shown in the results of Table 8, Level IV (low-risk) and Level I (extremely-high-risk) achieve relatively high values for all metrics. Precision, Recall, and F1-score decrease for Level III and Level II warning grades. The standard deviations of all experimental metrics are basically controlled within 3%, which demonstrates favorable robustness of the early-warning model on the Baijiabao landslide dataset.

## 4. Discussion

### 4.1. Ablation Experimental Results and Mechanism Analysis

#### 4.1.1. Discussion on Sliding-Window and Baseline Temporal Model Comparison

As shown in Table 3, the 90-day lookback window achieves optimal overall performance. Too-short windows cannot capture sufficient historical periodic evolutionary patterns, while excessively large windows introduce redundant samples and raise over-fitting risk.

As illustrated in Table 4 and Table 5, Transformer with larger parameter space tends to over-fit on our small-sample landslide dataset, whereas BiLSTM–Attention demonstrates stronger robustness for abrupt deformation events triggered by reservoir regulation and heavy rainfall.

#### 4.1.2. Mechanism Analysis of Gated-Attention Multimodal Ablation Experiments

**Figure 13:**
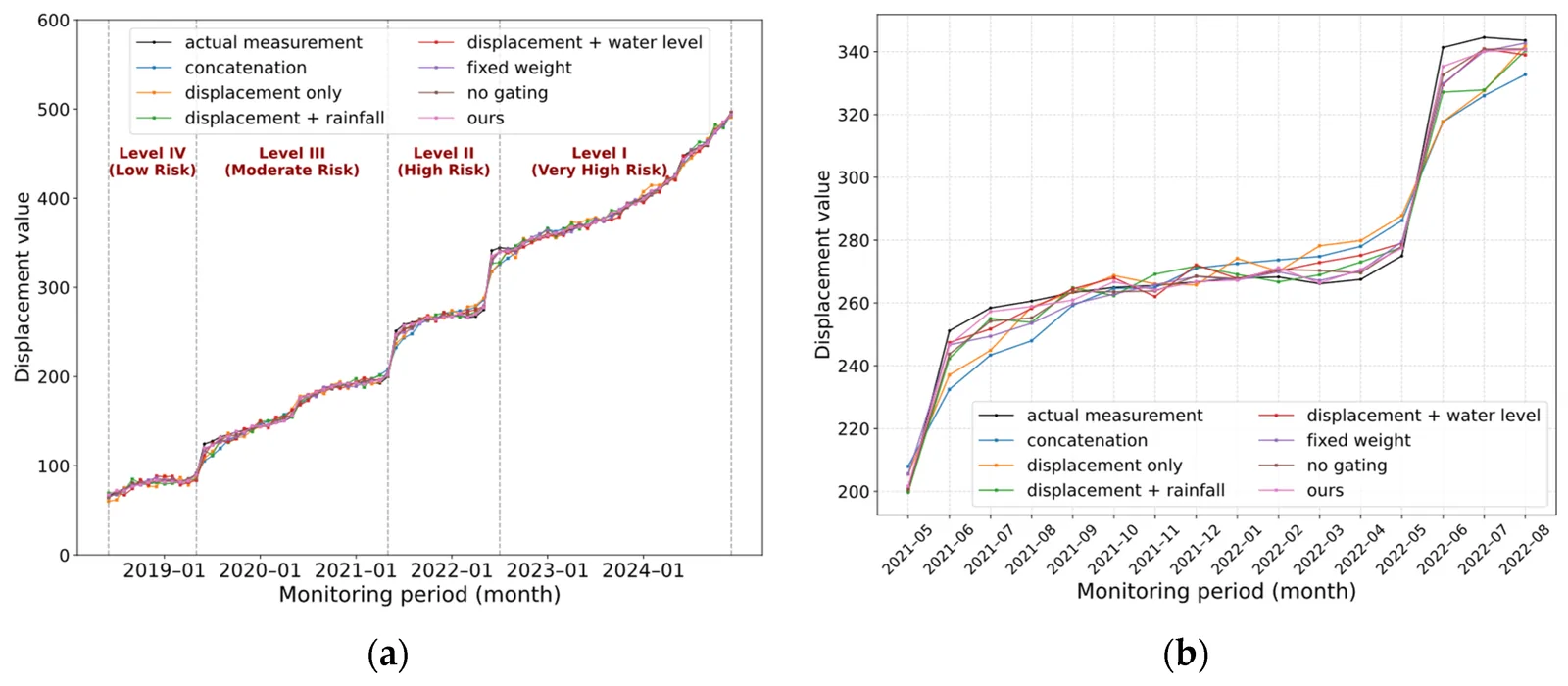
Comparison of landslide displacement prediction results from multimodal ablation experiments (1 d prediction horizon). (**a**) Full time series covering June 2018–December 2024; (**b**) Zoom-in view of a typical flood-season period (May 2021–August 2022).

**Figure 14:**
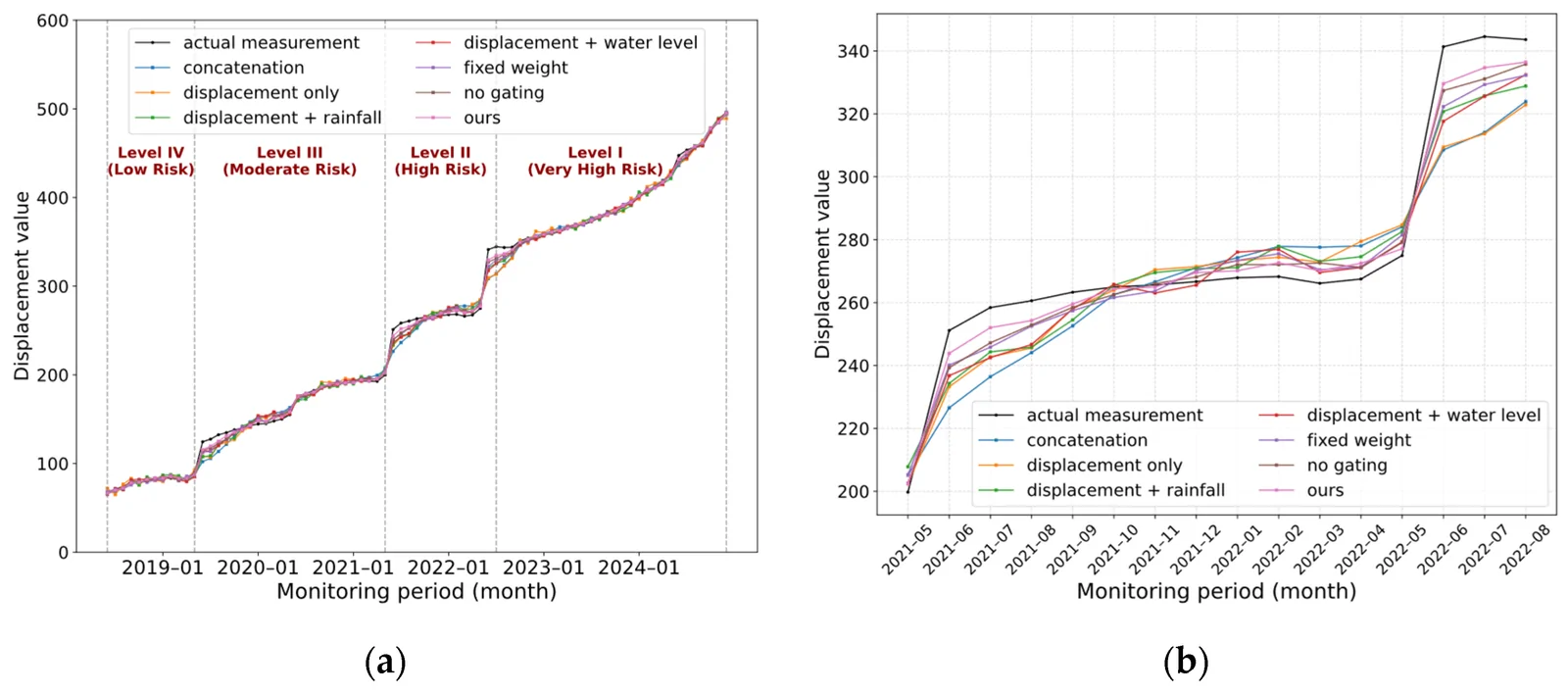
Comparison of landslide displacement prediction results from multimodal ablation experiments (7 d prediction horizon). (**a**) Full time series covering June 2018–December 2024; (**b**) Zoom-in view of a typical flood-season period (May 2021–August 2022).

Based on the quantitative ablation results of gated-attention multimodal fusion for landslide prediction in Table 6 (Section 3.5), together with the ablation time-series curves in Figure 13 and Figure 14, performance differences among various schemes are analyzed in this subsection.

When only single-modal displacement data are adopted, the model can fit the general trend during the stable deformation stage of landslides. Nevertheless, prediction errors increase remarkably under step-like deformation triggered by external disturbances such as rainfall and reservoir water level variation. Adding only one extra feature (either rainfall or reservoir water level) yields limited performance improvement. This confirms that landslide displacement is jointly driven by complex coupling effects between rainfall and reservoir water level. The absence of either feature will lead to the loss of partial valid information.

The direct concatenation of multimodal features may even produce larger errors than single-modal input. The underlying cause lies in information conflict or redundancy among features with distinct physical meanings from displacement, reservoir water level and rainfall. Simple concatenation cannot realize adaptive alignment among different modalities.

The fixed-weight scheme performs better than direct concatenation. However, its weights are globally optimized over the complete dataset. These static weights cannot adapt to dynamic shifts of dominant controlling factors during different creep stages or seasonal environmental changes. During steady-state creep and hydrologically stable periods, landslide displacement is mainly governed by the inertia of historical displacement. In flood seasons, heavy rainfall combined with rapid reservoir water level drawdown makes hydrological factors become dominant controlling factors. Global fixed weights fail to accommodate stage-dependent differences.

Further introducing the gating unit upon the attention mechanism can suppress interference caused by monitoring-sequence noise and outliers, and further improve model accuracy and robustness. This performance improvement is consistent with the mathematical formulation of gated-attention presented in Section 2.5.3: learnable gating coefficients serve as soft modality switches to suppress noisy modal inputs under different creep stages.

As illustrated by the time-series curves in Figure 13 and Figure 14, curves from different ablation models show similar overall trends for 1 d short-term prediction. Differences mainly appear at stages with step-like displacement jumps induced by flood-season heavy rainfall and reservoir operation. Short-term prediction presents relatively small yet frequently fluctuating errors, indicating that the model can rapidly respond to abrupt changes in rainfall and reservoir water level.

For 7 d medium-and-long-term prediction, overall discrepancies between ablation models increase significantly. Meanwhile, curve fluctuations are weaker than those seen in short-term prediction. Medium-and-long-term prediction integrates cumulative impacts from multi-day rainfall and reservoir water level changes. Therefore, gated-attention fusion plays a more important role in suppressing error accumulation. The proposed method can better capture displacement increments during step-like accelerated deformation, which is of practical significance for landslide early warning. Short-term prediction tests the model’s capability to respond to instantaneous disturbances, whereas medium-and-long-term prediction focuses on mitigating accumulated temporal errors. The gated-attention module works effectively under both scenarios, with more noticeable gains for medium-and-long-term prediction.

### 4.2. Interpretability Analysis of Gating Weights

**Figure 15:**
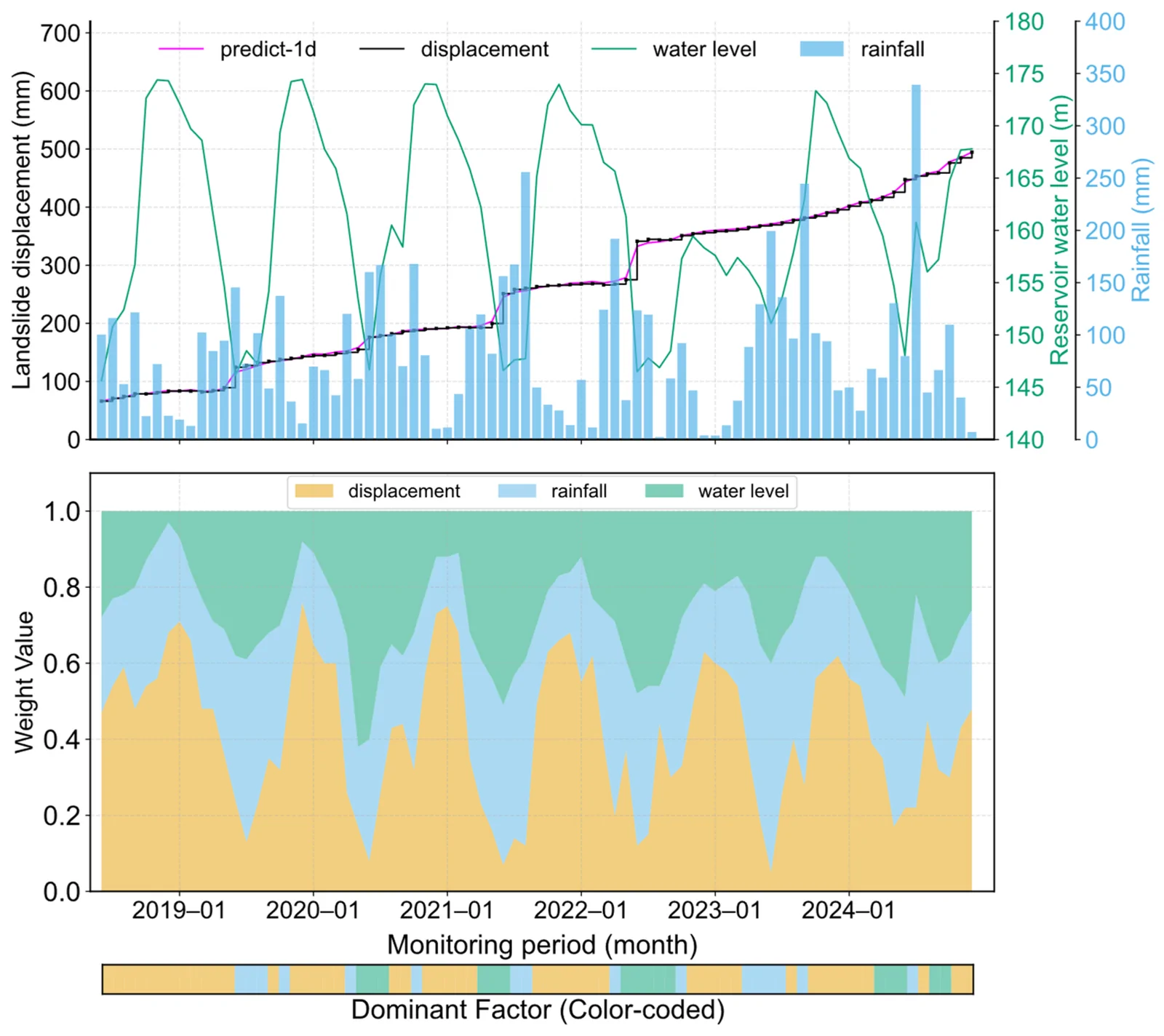
Temporal evolution diagram of multimodal-fusion gated weight coefficients (1 d prediction horizon).

To explore the dynamic evolution of fusion weights assigned to displacement, reservoir water level and rainfall modalities within the gated-attention module, dual-axis aligned subplots are employed. The upper subplot presents monitored time-series data and model-predicted displacement. The lower stacked subplot visualizes temporal variations in attention weights for each modality. The results are shown in Figure 15.

The height of each colored area in the stacked plot represents the contribution weight of the corresponding feature at a given moment. The sum of weights for the three features always equals one. Weights adjust dynamically along with evident periodic changes in reservoir water level and rainfall intensity. This observation demonstrates that the proposed gated-attention mechanism avoids static fixed weights.

During non-flood seasons (e.g., November to March of the next year), the reservoir maintains a relatively high and stable water level with low rainfall. The weight of displacement features remains at a high proportion. Under such conditions, external disturbances on landslides are weak, and landslide deformation is mainly driven by the inertia of historical deformation. The model relies more on historical GPS displacement time series for prediction, while weights for reservoir water level and rainfall are suppressed. This observation is consistent with the evolutionary law of creep stages.

In annual flood seasons (June–September), heavy rainfall coincides with reservoir water level drawdown, and landslide hazards occur frequently. As shown in the lower stacked plot of Figure 15, weights corresponding to rainfall and reservoir water level increase synchronously, while the weight of displacement features decreases accordingly. It indicates that gated attention can dynamically adjust the contribution proportion of each modal feature according to external environmental conditions. Thus, the model can capture step-like displacement increments induced by external landslide-triggering factors. For instance, flood seasons with extreme heavy rainfall and rapid reservoir drawdown in 2021 and 2022 are highly consistent with periods of accelerated landslide displacement in time.

Traditional feature concatenation or fixed-weight fusion assigns constant feature importance over the whole time span and cannot identify shifts among dominant landslide-triggering factors. The proposed gated attention realizes adaptive dynamic weight adjustment and improves prediction and early-warning adaptability across different landslide evolution stages. Nevertheless, drastic weight fluctuations are observed in a few time intervals. These fluctuations are presumed to originate from monitoring noise, complex coupling between rainfall and water level, and external forces such as local construction. Further investigation will be carried out with more landslide monitoring samples and additional modal features.

### 4.3. Analysis of the Role of Fine-Grained Rainfall Gridding Preprocessing

In this work, PSO–Kriging interpolation combined with concentric-ring gridding is adopted to generate 21-dimensional rainfall feature vectors, achieving rainfall representation at a 500 m spatial resolution. This approach differs from conventional landslide prediction studies that directly take single rain-gauge records as input. Single-point rain-gauge data only reflect local rainfall near the station and fail to capture rainfall spatial heterogeneity within the 0.5–5 km surrounding area of landslides.

By calculating maximum and average rainfall over multiple concentric rings, both rainfall information at the central site and spatial rainfall differences in surrounding regions are preserved. Consequently, rainfall feature vectors better matching the actual geological environment of the reservoir area are provided for the model. According to overall experimental results, fine-grained rainfall input serves as an essential prerequisite for the multimodal model to accurately reproduce step-like landslide deformation in flood seasons. If only single-point rain-gauge data are used, local heavy rainfall tends to be neglected, which enlarges prediction errors during accelerated-deformation periods.

Horizontal comparison is conducted against published deep-learning-based landslide prediction studies for the TGRA. Most existing LSTM- or BiLSTM-related works directly employ single-point rain-gauge data and apply static feature fusion strategies, generally yielding large prediction errors during step-like accelerated deformation stages [19,22]. This work adopts PSO–Kriging-based multi-ring fine-grained rainfall representation together with the gated-attention dynamic multimodal-fusion mechanism. As reported in Table 6 and Table 8, our proposed framework obtained competitive prediction and warning performance for TGRA landslides across multi-scale prediction horizons, when compared with performance metrics reported in published LSTM-based or BiLSTM-based landslide prediction studies for this region.

Unlike most existing studies that only implement displacement regression, the proposed framework additionally realizes four-level multi-level early warning. Studies differ in landslide samples, dataset partitioning, and prediction horizons. For this reason, the above accuracy metrics serve only as references and cannot directly quantify performance gains. Even so, these results illustrate the practical value of our framework for engineering-oriented early warning.

It should be noted that medium-and-long-term (7 d–15 d) prediction relies heavily on external CMA rainfall forecast inputs. As shown in Supplementary Table S1, rainfall forecast errors grow as forecast lead time extends. These meteorological uncertainties propagate into displacement prediction results. Therefore, the total prediction error for 7 d and 15 d outputs contains two components: inherent error from meteorological forecasting, and residual error originating from the landslide prediction model itself. This distinction is critical when interpreting the model’s practical performance for medium–long-term warning.

### 4.4. Event-Oriented Performance Analysis for Accelerated-Deformation Events

Overall averaged regression metrics such as RMSE and MAE reflect global statistical prediction accuracy. Nevertheless, favorable average numerical performance does not directly equal practical engineering early-warning capability. From hazard-mitigation perspective, model behavior during high-risk accelerated-deformation episodes triggered by heavy rainfall, rapid reservoir drawdown and displacement jumps carries higher practical significance than average performance calculated over long stable time periods.

We selected two representative hazard-relevant event windows from the Baijiabao independent test set: Event 1 (May–August 2021, flood-season heavy rainfall coupled with reservoir drawdown, initial acceleration stage); Event 2 (April–July 2022, prominent step-like displacement jump entering intermediate-acceleration stage). Event-specific prediction and warning metrics are calculated for these two critical periods, as summarized in Supplementary Table S3.

Compared with global test-set averaged metrics, prediction errors rise distinctly during these accelerated-deformation hazard episodes. This phenomenon reveals that, despite satisfactory overall numerical accuracy, the model still faces larger forecasting challenges during rapid-deformation events which matter most for early warning. This event-based evaluation highlights the inherent gap between global statistical accuracy and real-world engineering usefulness, and is consistent with the study limitations discussed in Section 5.

## 5. Conclusions

Focusing on the engineering challenge of landslides induced by the coupling of local heavy rainfall and reservoir water level fluctuations in the TGRA, this study integrates multi-source sensing time-series data, including GPS displacement, reservoir water level sensors, ground meteorological stations, weather radar, and multi-timescale meteorological forecast maps. A multi-task framework for landslide displacement prediction and multi-level early warning is constructed. The framework integrates multiple modules: PSO–Kriging-based spatial rainfall reconstruction, fine-grained rainfall gridding, multi-branch BiLSTM–Attention temporal feature extraction, and gated-attention adaptive multimodal fusion.

PSO–Kriging interpolation provides high-quality spatial rainfall inputs for landslide prediction. Compared with conventional LSTM and BiLSTM, BiLSTM–Attention exhibits stronger capability to capture step-like jumps of reservoir water level regulation and landslide displacement. The gated-attention mechanism adaptively fuses multimodal features, and dynamic gating weights show favorable interpretability consistent with landslide geological evolution laws. This work realizes multi-scale landslide displacement regression prediction from 1 d to 15 d. The macro-average F1-score reaches 89.3 ± 1.9% for 1 d short-term early-warning and 81.7 ± 1.2% for 7 d medium-and-long-term early-warning. Furthermore, a four-level early-warning mechanism is established based on the five-stage creep theory of landslides. It supplies multi-level decision support for TGRA administrators, ranging from emergency response to medium-and-long-term trend assessment.

This research still has several limitations. The model is mainly validated on landslides within the TGRA. Its generalization performance for landslides under other geological, meteorological, and hydrological conditions requires further verification with more field-measured datasets. Only surface time-series sensing and meteorological rainfall forecast data are adopted as inputs, making it difficult to capture early damage signals inside slope masses. Restricted by natural geological-hazard sampling conditions, high-risk samples of complete landslide failure and samples under extreme multi-factor coupling are insufficient. Local oscillations of gated-attention weights occur in certain periods, and engineering deployment on edge terminals has not yet been completed.

Future work will improve the framework from the following aspects. Landslide monitoring datasets with diverse geological backgrounds will be supplemented. Weight-smoothing regularization constraints will be introduced to suppress abnormal weight fluctuations. DEM terrain data, UAV imagery, and underground sensing monitoring data will be fused to incorporate topographic and geomorphic features and improve identification performance during landslide accelerated-deformation stages. Weight-smoothing regularization will mitigate local weight oscillations. The model will be further lightweighted to adapt to low-power edge devices in the reservoir area and promote practical engineering deployment of the proposed algorithm.

## Figures and Tables

**Table 1:** Cross-validation comparison among different interpolation algorithms.

Interpolation Method	ME (mm)	MAE (mm)	RMSE (mm)
IDW	2.34 ± 0.54	2.96 ± 0.45	5.85 ± 0.82
Ordinary Kriging	0.61 ± 0.22	1.42 ± 0.19	2.83 ± 0.37
PSO–Kriging	0.49 ± 0.13	0.97 ± 0.25	1.65 ± 0.24

**Table 2:** Comparison of variogram parameters and interpolation errors between manual fitting and PSO optimization.

Parameter Estimation Method	*C*_0_	*C*	Range (km)	ME	MAE	RMSE
Manual Fitting (Ordinary Kriging)	2.72	10.48	37.4	0.61 ± 0.22	1.42 ± 0.19	2.83 ± 0.37
PSO Optimization (PSO–Kriging)	1.06	12.35	52.8	0.49 ± 0.13	0.97 ± 0.25	1.65 ± 0.24

**Table 3:** Five-fold cross-validation results of reservoir water level and landslide displacement prediction under different sliding-window sizes.

Type	Metric	30 Day	60 Day	90 Day	180 Day	365 Day
	ME	1.26 ± 0.23	−0.11 ± 0.15	−0.12 ± 0.08	0.13 ± 0.11	0.41 ± 0.32
Reservoir Water Level (m)	MAE	2.85 ± 0.37	1.56 ± 0.41	0.84 ± 0.31	1.04 ± 0.43	1.66 ± 0.53
	RMSE	3.61 ± 0.68	2.09 ± 0.62	1.65 ± 0.46	1.58 ± 0.39	2.07 ± 0.85
	ME	2.28 ± 2.63	−1.67 ± 2.15	0.66 ± 0.98	0.73 ± 1.15	1.49 ± 2.86
Landslide Displacement (mm)	MAE	6.32 ± 2.74	4.28 ± 2.09	3.54 ± 1.63	3.97 ± 1.80	5.13 ± 2.63
	RMSE	8.07 ± 2.99	5.32 ± 2.56	4.93 ± 1.96	5.86 ± 2.34	7.38 ± 3.34

**Table 4:** Comparison of multi-scale reservoir water-level prediction results among various temporal models (unit: m).

Model	Metric	1 d	2 d	3 d	7 d	15 d
	ME	0.94 ± 1.01	−0.22 ± 1.39	−0.46 ± 2.08	−1.79 ± 3.25	−2.18 ± 2.89
LSTM	MAE	1.82 ± 1.12	2.79 ± 1.51	3.55 ± 3.66	3.83 ± 3.63	6.45 ± 3.72
	RMSE	2.85 ± 2.03	3.59 ± 2.72	4.06 ± 2.61	5.34 ± 3.75	8.71 ± 4.78
	ME	0.71 ± 0.73	0.95 ± 0.91	0.63 ± 1.08	0.97 ± 1.39	1.49 ± 2.16
BiLSTM	MAE	1.19 ± 0.74	1.83 ± 0.98	2.16 ± 1.74	3.97 ± 1.80	3.44 ± 1.64
	RMSE	2.37 ± 2.35	3.91 ± 2.87	3.85 ± 3.12	5.86 ± 3.34	5.97 ± 3.42
	ME	−0.12 ± 0.08	−0.31 ± 0.17	−0.54 ± 0.43	−0.72 ± 0.38	−1.18 ± 0.74
BiLSTM–Attention	MAE	0.84 ± 0.31	1.91 ± 0.56	2.66 ± 1.06	3.04 ± 2.13	3.25 ± 1.83
	RMSE	1.65 ± 0.46	2.62 ± 1.04	2.97 ± 1.61	4.36 ± 2.76	4.55 ± 2.13
	ME	−0.17 ± 0.06	−0.19 ± 0.17	−0.73 ± 0.85	−0.48 ± 0.31	−1.05 ± 0.91
Transformer	MAE	0.92 ± 0.30	2.73 ± 0.85	2.56 ± 1.52	3.19 ± 1.75	3.56 ± 1.38
	RMSE	1.74 ± 0.51	2.91 ± 0.94	4.12 ± 1.75	4.79 ± 2.63	5.12 ± 1.95

**Table 5:** Comparison of multi-scale landslide displacement prediction results among various temporal models (unit: mm).

Model	Metric	1 d	2 d	3 d	7 d	15 d
	ME	−0.89 ± 2.86	−1.85 ± 2.64	−1.25 ± 3.65	2.92 ± 4.81	−3.27 ± 6.38
LSTM	MAE	6.55 ± 3.45	9.58 ± 3.23	9.83 ± 2.93	10.58 ± 3.23	11.75 ± 5.92
	RMSE	9.02 ± 3.48	10.97 ± 4.16	12.31 ± 4.76	14.18 ± 5.32	15.80 ± 6.93
	ME	0.71 ± 0.73	−1.10 ± 1.76	−1.63 ± 2.88	2.41 ± 3.96	3.57 ± 4.67
BiLSTM	MAE	3.49 ± 1.84	3.80 ± 2.89	6.86 ± 1.74	8.63 ± 3.01	11.30 ± 5.31
	RMSE	5.37 ± 2.35	5.91 ± 3.32	9.58 ± 4.12	11.04 ± 3.34	13.68 ± 6.57
	ME	0.66 ± 0.98	−1.10 ± 1.76	−0.25 ± 2.16	−2.14 ± 2.93	−2.29 ± 4.32
BiLSTM–Attention	MAE	3.54 ± 1.63	4.33 ± 3.18	4.57 ± 2.92	7.67 ± 4.05	11.02 ± 4.05
	RMSE	4.93 ± 1.96	5.69 ± 2.42	6.82 ± 2.75	10.36 ± 4.18	13.25 ± 6.30
	ME	−0.49 ± 1.08	−0.51 ± 1.93	1.28 ± 2.10	−1.53 ± 3.15	−1.29 ± 3.81
Transformer	MAE	3.83 ± 2.81	4.76 ± 3.34	5.82 ± 3.71	9.01 ± 3.84	11.67 ± 5.86
	RMSE	5.64 ± 2.42	7.54 ± 4.13	8.06 ± 4.08	10.85 ± 2.96	14.03 ± 4.92

**Table 6:** Ablation experimental results of landslide prediction using gated-attention multimodal fusion (90-day sliding window; displacement unit: mm).

Model	ME (1 d)	MAE (1 d)	RMSE (1 d)	ME (7 d)	MAE (7 d)	RMSE (7 d)
Displacement Only	0.66 ± 0.98	3.54 ± 1.63	4.93 ± 1.96	−2.14 ± 2.93	7.67 ± 4.05	10.36 ± 4.18
Displacement + Rainfall	0.49 ± 0.73	3.39 ± 1.82	4.72 ± 2.15	−1.83 ± 2.71	6.92 ± 3.80	9.83 ± 4.06
Displacement + Water level	−0.25 ± 1.07	3.31 ± 1.49	4.45 ± 2.27	0.59 ± 2.67	6.84 ± 3.15	10.17 ± 3.73
Concatenation	1.05 ± 1.28	4.61 ± 2.01	5.13 ± 2.48	−1.47 ± 3.26	8.06 ± 3.57	11.04 ± 3.95
Fixed Weight	0.87 ± 1.13	3.13 ± 1.82	4.42 ± 2.06	−1.61 ± 2.03	6.02 ± 3.16	8.62 ± 3.71
No Gating	0.79 ± 1.35	2.96 ± 1.37	3.98 ± 1.84	−1.19 ± 2.25	5.89 ± 2.55	7.73 ± 3.58
Ours (Gated-Attention Fusion)	−0.37 ± 0.62	2.55 ± 1.18	3.42 ± 1.27	−1.06 ± 1.77	5.12 ± 2.61	7.08 ± 3.21

**Table 7:** Quantitative reproducible labeling criteria for four-level warning labels.

Warning Level	Creep Stage	${\bar{v}}_{\mathrm{win}7}$	S-t Curve Morphological Feature	Auxiliary Rule for Boundary Samples
Level IV (Low Risk)	Initial deformation Constant-velocity	${\bar{v}}_{\mathrm{win}7}$ < 0.55	Slow near-linear displacement growth; low curvature	Near 0.55 mm/d: low cumulative rainfall and stable reservoir water level
Level III (Moderate Risk)	Initial acceleration	0.5 ≤ ${\bar{v}}_{\mathrm{win}7}$ < 2.1	Gradually increasing curvature; moderate speed rise	Boundary samples: rising rainfall/reservoir drawdown confirms initial acceleration
Level II (High Risk)	Intermediate acceleration	2.1 ≤ ${\bar{v}}_{\mathrm{win}7}$ < 5.8	Distinct upward curvature; continuous accelerating deformation	Boundary samples: sustained heavy rainfall/rapid reservoir drawdown supports high-risk label
Level I (Very High Risk)	Critical instability	${\bar{v}}_{\mathrm{win}7}$ > 5.8	Sharp exponential-like rise; approaching catastrophic failure	Boundary samples: extreme rainfall + rapid water-level drop indicates imminent failure

**Table 8:** Performance of the multi-level early-warning model on the Baijiabao landslide test set for 1-day and 7-day ahead forecasts (%).

Warning Level	Creep Stage	1-Day Ahead Forecast			7-Day Ahead Forecast		
		Precision	Recall	F1-Score	Precision	Recall	F1-Score
Level IV (Low Risk)	Initial deformation Constant-velocity	93.2 ± 1.4	94.7 ± 0.9	93.9 ± 1.1	85.6 ± 2.4	87.1 ± 1.6	86.3 ± 1.4
Level III (Moderate Risk)	Initial acceleration	87.4 ± 2.3	84.8 ± 2.8	86.1 ± 2.5	78.9 ± 2.7	76.3 ± 2.5	77.6 ± 1.9
Level II (High Risk)	Intermediate acceleration	81.5 ± 3.2	83.6 ± 2.1	82.5 ± 2.6	71.7 ± 3.7	73.5 ± 2.2	72.6 ± 2.2
Level I (Very High Risk)	Critical instability	92.9 ± 1.8	96.5 ± 1.2	94.7 ± 1.4	89.2 ± 2.1	90.6 ± 1.8	89.9 ± 1.4
All Levels	All stages	88.7 ± 2.2	89.9 ± 1.8	89.3 ± 1.9	81.5 ± 1.4	81.8 ± 1.1	81.7 ± 1.2

## Data Availability

Landslide monitoring time-series data (including displacement, reservoir water level and point rainfall records, 2007–2024) used in this study were obtained from the National Cryosphere Desert Data Center of China (https://www.ncdc.ac.cn/). Weather-radar echo maps and multi-lead-time meteorological forecast maps for short-term and medium-and-long-term rainfall analysis were acquired from the National Meteorological Center of China (https://nmc.cn/). Restrictions apply to the redistribution of these third-party raw observational data; interested researchers need to obtain permission from the above-mentioned institutions for access.

## Supplementary Materials

The following supporting information can be downloaded at https://www.mdpi.com/article/10.3390/s26196207/s1: Table S1: CMA multi-lead-time rainfall forecast validation (ground rain gauge as ground truth, unit: mm); Table S2: Comparison of trainable parameters and computational complexity among the four temporal models; Table S3: Event-based evaluation metrics for critical accelerated-deformation event windows on Baijiabao independent test set.

## Abbreviations

The following abbreviations are used in this manuscript:

BiLSTM	Bidirectional Long Short-Term Memory
CMA	China Meteorological Administration
FLOPs	Floating Point Operations
GPS	Global Positioning System
GNSS	Global Navigation Satellite System
IDW	Inverse Distance Weighting
InSAR	Interferometric Synthetic Aperture Radar
LSTM	Long Short-Term Memory
MAE	Mean Absolute Error
ME	Mean Error
PSO	Particle Swarm Optimization
RMSE	Root Mean Square Error
SVR	Support-Vector Regression
TGRA	Three Gorges Reservoir Area
