# Supplementary material for: Multi-Scale Landslide Displacement Prediction and Multi-Level Early Warning for the Three Gorges Reservoir Area Using Multi-Source Sensing and Gated-Attention Multimodal Fusion
Source: Sensors (Basel). 2026 Sep 30;26(19):6207. doi: 10.3390/s26196207 (PMC13667668; doi:10.3390/s26196207)
Supplement: Supplementary file 1 [file sensors-26-06207-s001.zip › sensors-4560949-supplementary.pdf]

sensors

MDPI

Supplementary Materials

Multi-Scale Landslide Displacement Prediction and Multi-Level Early Warning for the Three Gorges Reservoir Area Using Multi-Source Sensing and Gated-Attention Multimodal Fusion

Liangwu Xu [1,*], Xiangjin Ran [2], Lili Yao [1] and Zhaoji Lin [1]

[1] School of Computer Science and Engineering, Sanjiang University, Nanjing 210012, China; yao_lili@sju.edu.cn (L.Y.); lin_zhaoji@sju.edu.cn (Z.L.)
[2] College of Earth Sciences, Jilin University, Changchun 130061, China; ranxiangjin@jlu.edu.cn
* Correspondence: xu_liangwu@sju.edu.cn

This supplementary document contains Supplementary Tables S1–S3. Supplementary Table S1 provides CMA multi-lead-time rainfall forecast validation against ground rain-gauge observations in the Three Gorges Reservoir Area; Supplementary Table S2 reports the trainable parameters and computational complexity among the four temporal prediction models; and Supplementary Table S3 provides event-based evaluation metrics for critical accelerated-deformation event windows on the Baijiabao independent test set.

Table S1. CMA multi-lead-time rainfall forecast validation (ground rain-gauge as ground-truth, unit: mm).

Forecast Lead Time (h)	ME (mm)	MAE (mm)	RMSE (mm)
24	0.22±0.09	0.68±0.21	1.35±0.20
48	0.35±0.13	1.04±0.25	1.67±0.26
72	0.49±0.17	1.29±0.30	2.01±0.29
96	0.61±0.19	1.54±0.33	2.37±0.32
120	0.73±0.21	1.60±0.35	2.82±0.37
144	0.89±0.25	1.82±0.38	3.06±0.41
168	0.93±0.26	1.96±0.41	3.31±0.43

Note: Validation is performed on 583 rain-gauge stations within Three Gorges Reservoir Area; metrics are reported as mean ± standard deviation from five-fold cross-validation.

Table S2. Comparison of trainable parameters and computational complexity among the four temporal models.

Model	Hidden Size	Layers	Attention Header	Trainable Params (M)	FLOPs (G)	Training Time (s/epoch)	Inference Time (ms/sample)
LSTM	128	2	-	0.41	1.79	11.7	1.19
BiLSTM	128	2	-	0.85	3.72	20.6	2.27
BiLSTM–Attention	128	2	4	1.09	4.85	27.2	2.83
Transformer	128	4	8	3.21	18.03	69.8	8.46

Note: FLOPs are calculated for a single forward pass with input sequence length = 90. Training/inference time is measured on an NVIDIA RTX 3090 GPU with batch size = 1. Hidden Size denotes the number of neurons within each hidden layer.

Table S3. Event-based evaluation metrics for critical accelerated-deformation event windows on Baijiabao independent test set.

Event ID	Time Window	Event type	Deformation Stage	1-d RMSE (mm)	1-d Warning F1-score (%)	7-d RMSE (mm)	7-d Warning F1-score (%)
Event 1	May-Aug 2021	Heavy rainfall + reservoir drawdown	Initial acceleration	4.11	79.1	7.83	73.4
Event 2	Apr-Jul 2022	Step-like displacement jump	Intermediate acceleration	5.28	74.6	9.49	70.2

Note: Global test-set baseline: 1-d RMSE =3.42 ± 1.27 mm, warning macro F1-score = 89.3 ± 1.9 %; 7-d RMSE =7.08±3.21 mm, warning macro F1-score =81.7 ± 1.2 %. Event-based metrics are calculated for individual fixed deformation windows, so no standard deviation is available for each event.
